# Susceptibilities of CNS Cells towards Rabies Virus Infection Is Linked to Cellular Innate Immune Responses

**DOI:** 10.3390/v15010088

**Published:** 2022-12-29

**Authors:** Lena Feige, Tatsuya Kozaki, Guilherme Dias de Melo, Vincent Guillemot, Florence Larrous, Florent Ginhoux, Hervé Bourhy

**Affiliations:** 1 Institut Pasteur, Université de Paris, Lyssavirus Epidemiology and Neuropathology, 75015 Paris, France; 2 Singapore Immunology Network, Agency for Science, Technology and Research, 8A Biomedical Grove, Immunos Building, Level 3, Singapore 138648, Singapore; 3 Hub de Bioinformatique et Biostatistique, Département Biologie Computationnelle, Institut Pasteur, 75015 Paris, France; 4 Shanghai Institute of Immunology, Shanghai Jiao Tong University School of Medicine, 280 South Chongqing Road, Shanghai 200025, China; 5 Translational Immunology Institute, SingHealth Duke-NUS Academic Medical Center, 20 College Road, Discovery Tower Level 8, Singapore 169856, Singapore; 6 Inserm U1015, Gustave Roussy, Bâtiment de Médecine Moléculaire, 114 Rue Edouard Vaillant, 94800 Villejuif, France

**Keywords:** rabies, astrocytes, microglia, neurons, viral tropism, immune evasion

## Abstract

Rabies is caused by neurotropic rabies virus (RABV), contributing to 60,000 human deaths annually. Even though rabies leads to major public health concerns worldwide, we still do not fully understand factors determining RABV tropism and why glial cells are unable to clear RABV from the infected brain. Here, we compare susceptibilities and immune responses of CNS cell types to infection with two RABV strains, Tha and its attenuated variant Th2P-4M, mutated on phospho- (P-protein) and matrix protein (M-protein). We demonstrate that RABV replicates in human stem cell-derived neurons and astrocytes but fails to infect human iPSC-derived microglia. Additionally, we observed major differences in transcription profiles and quantification of intracellular protein levels between antiviral immune responses mediated by neurons, astrocytes (*IFNB1*, *CCL5*, *CXCL10*, *IL1B*, *IL6*, and *LIF*), and microglia (*CCL5*, *CXCL10*, *ISG15*, *MX1*, and *IL6*) upon Tha infection. We also show that P- and M-proteins of Tha mediate evasion of NF-κB- and JAK-STAT-controlled antiviral host responses in neuronal cell types in contrast to glial cells, potentially explaining the strong neuron-specific tropism of RABV. Further, Tha-infected astrocytes and microglia protect neurons from Tha infection via a filtrable and transferable agent. Overall, our study provides novel insights into RABV tropism, showing the interest in studying the interplay of CNS cell types during RABV infection.

## 1. Introduction

Rabies is caused by RABV, a negative-sense, single-stranded RNA virus belonging to the family *Mononegavirales* [1]. RABV belongs to the genus *Lyssavirus*, family *Rhabdoviridae*, and presents an RNA genome 12 kb in length: the viral genome encodes nucleoprotein (N-protein), phosphoprotein (P-protein), matrix protein (M-protein), glycoprotein (G-protein), and the large protein (L-protein). RABV, classically believed to present a neuron-specific tropism, reaches the CNS via retrograde transport along the neural network, where it induces fatal encephalomyelitis in mammals, including humans [2,3]. Once inside the CNS, RABV successfully hides inside the neural network from glial surveillance [4]. To date, we do not fully understand the exact mechanisms underlying viral-mediated immune evasion of glial cells.

Cellular tropism relies on two major determinants: the expression of entry receptors, which enables viral entry, and the cellular immune response, which allows or restricts productive viral replication [5]. In detail, the innate immune system represents the first line of defense against viral invaders. It senses viruses via germline-encoded pattern recognition receptors (PRRs), including toll-like receptors (TLRs), retinoic acid-inducible gene I (*RIG-I*) like helicases (RLRs), and nucleotide-binding oligomerization domain-like *receptors* (*NOD-like receptors*). TLR3 and RLRs in the host cells recognize virus-derived RNAs, leading to the activation of transcription factors, more specifically interferon regulatory factor 3 (IRF-3) and nuclear factor κB (NF-κB), which establish antiviral responses via the production of type I IFNs and proinflammatory cytokines [6]. Subsequently, type I IFNs bind type I IFN receptors (IFNAR) and activate the Janus kinase (JAK) and signal transducer and activator of the transcription protein 1 (STAT1) signaling pathway, elevating the expression of interferon-stimulated genes (ISGs) with antiviral activity.

RABV uses several receptors to enter cells via clathrin-mediated endocytosis [7,8,9]: nicotinic acetylcholine receptor (nAChR) [10], neuronal cell adhesion molecule (NCAM) [11], low-affinity p75 neurotrophin receptor (p75NTR) [12], and metabotropic glutamate receptor subtype 2 (mGluR2) [13]. However, those broadly expressed receptors are not essential for RABV entry per se [14] but lead to an acceleration of RABV infection [15]. In contrast to its profound neuron-specific tropism in vivo [16,17], most cell types are susceptible to RABV infection in vitro [18,19,20]. Whereas most of the research is focussed on the discovery of RABV receptors [10,11,12,13] and their interaction with the RABV G-protein [14], less research focusses on how cellular host immune responses shape RABV tropism and how distinct neural immune responses in the CNS could collectively lead to the establishment of an antiviral response. Recently, several publications reported infection of different glial cells in vivo, particularly astrocytes [21,22] and Schwann cells [23], depending on the viral strain and the infection route used [24]. Nevertheless, we are far from understanding the molecular pathways underlying susceptibility to RABV infection, although it remains crucial to determine infection outcome.

Here, we investigated susceptibilities and cellular immune responses of different CNS cell types towards infection with two canine RABV strains (Tha and Th2P-4M) in vitro. Tha is a cell culture-adapted virus isolate [25] that shares the same genetic background with Th2P-4M, except for mutations introduced into P- (W265G andM287V) and M-proteins (R77K, D100A, A104S, and M110L), consequently inhibiting viral evasion of the NF-κB and JAK-STAT pathways [26,27,28,29]. We provide evidence that virulent Tha and less virulent Th2P-4M successfully replicate in hiNeurons and, to a lesser extent, in hiAstrocytes but not in hiMicros. Further, Tha strongly represses innate immune gene expression and the secretion of inflammatory proteins in neurons in contrast to glial cell types. Whereas successful infection might highly depend on the concentration of RABV receptors on the cellular surface, we suggest that cell type-specific innate immune responses are critical for the success of RABV replication and spread in the distinct CNS cell types. Hence, our study emphasizes the need to study RABV in relevant CNS culture models to understand the underlying pathways shaping RABV tropism as well as to elucidate the role of glial cells in the RABV-infected brain.

## 2. Materials and Methods

### 2.1. Viruses

Thailand virus, referred to as 8764THA (Genbank No. EU293111) is a field strain of RABV isolated from the brain of a Thai patient who died of rabies after being bitten by a rabid dog [25]. This virus was further adapted to cell culture on BSR cells (a BHK-21 clone, kindly provided by Monique Lafon, Institute Pasteur, Paris) [30], consequently called 8743THA (Genbank No. EU293121, EVAg collection, Ref-SKU: 014V-02106) [25] and in this manuscript referred to as Tha. Sequence comparison between the cell culture-adapted RABV strain 8743THA (GenBank No. EU293121) and the original field isolate 8764THA (GenBank No. EU293111) demonstrated 98.56% identity (52 mutations) and 99.36% similarity between concatenated protein sequences. The recombinant Th2P-4M virus harbours the same genetic background as Tha apart from bearing two mutations in the viral P-protein (W265G and M287V) and four mutations in the viral M-protein (R77K, D100A, A104S, and M110L), which were previously shown to inhibit evasion of the NF-κB and JAK-STAT pathways [26,31,32]. To monitor viral infection, recombinant viral Tha-eGFP and Th2P-4M-eGFP constructs were used, which were generated by cloning sequences of eGFP (recovered from pEGFP-C1 plasmid, Promega, Charbonnières-les-Bains, France) into the genetic sequence of Tha [33] and Th2P-4M, respectively. Viral strains were sequenced before being used for infection experiments. Viral titres were determined by virus titration on BSR cells, more specifically via staining of the viral nucleoprotein (5100, Sigma-Aldrich, Saint-Quentin-Fallavier Cedex, France). No significant difference was observed in the number of viral particles detected in the supernatant of Tha and Th2P-4M-infected BSR-T7 cells (a BHK-21 clone) [26]. Further, the impact of introduced mutations into viral P- and M-proteins was shown to have no impact on viral replication (Figure S1).

### 2.2. Cell Culture and Infection

The human neuroblastoma cell line SK-N-SH (ATCC^®^ HTB-11™), the human astrocyte-like cell line SVGp12 (ATCC^®^ CRL8621™), and the human microglial cell line HMC3 (ATCC^®^ CRL-3304^™^) were cultured in Dulbecco’s Modified Eagle Medium (10566016, Thermo Scientific, Illkirch-Graffenstaden, France) supplemented with 10% heat-inactivated foetal bovine serum (FCS, S182H-500, Eurobio Scientific, Les Ulis, France) at 37 °C and 5% CO_2_.

Human neuronal progenitor cells (EnStem-A™ SCC003, Merck Millipore, Molsheim, France) were cultured on plates previously coated with Geltrex (A1413201, Invitrogen, Waltham, MA, USA) in complete neural stem cell (CNSC) medium containing Knock-out D-MEM/F-12 (A1370801, Thermo Scientific), 2 mM Glutamax (35050038, Thermo Scientific), 2% StemPro Neural Supplement (A1050801, Thermo Scientific), 20 ng/mL FGF (PHG0026, Thermo Scientific), and 20 ng/mL EGF (PHG0311, Thermo Scientific) for amplification purposes at 37 °C and 5% CO_2_.

Human foetal pAstrocytes (N7805100, Thermo Scientific) were cultured on Geltrex-coated plates (A1413201, Thermo Scientific) in complete astrocyte medium containing D-MEM (10566016, Thermo Scientific), 2% foetal bovine serum (S182H-500, Eurobio), and N-2 Supplement (17502048, Thermo Scientific) at 37 °C and 5% CO_2_.

Human iPSCs (XCL-1, IP-001-1V, XCell Science) were cultured on Matrigel-coated plates (354277, Corning, Amsterdam, The Netherlands) in mTeSR1 medium (85850, STEMCELL Technology, Saint-Égrève, France). The medium was changed daily.

Upon 80% confluency, cells were used for infection experiments. The cell medium was aspirated, and cells were washed once with PBS (10010023, Thermo Scientific) prior to infection. Adjacently, Tha and Th2P-4M were diluted in culture medium according to the appropriate multiplicity of infection (MOI). Cells were incubated with viral suspension for 2 h at 37 °C and 5% CO_2_. After two hours, the viral suspension was removed, and the appropriate culture medium was added. Twenty-four hours post infection, cells were treated with 2500 U/mL of IFN-α (I4401-100KU, Sigma-Aldrich) and incubated for 24 h at 37 °C and 5% CO_2_ prior to cell lysis.

### 2.3. Differentiation of hNSC to hiNeurons

To induce neural differentiation, EnStem-A cells were cultured at a density of 5 × 10^4^ cells/cm^2^ on plates previously coated with Geltrex (A1413302, Thermo Scientific) according to the manufacturer’s instructions. One day after seeding, CNSC was changed to a neural differentiation medium (NDM) consisting of neurobasal medium (10880022, Thermo Scientific), B-27 Serum Free Supplement (17504044, Thermo Scientific), 2 mM GlutaMAX (35050038, Thermo Scientific), CultureOne Supplement (A33202-01, Thermo Scientific), and 200 μM ascorbic acid (A4403, Sigma-Aldrich). After 21 days of differentiation, differentiated neurons were used for downstream experiments. 

### 2.4. Differentiation of hiPSCs to hiMacs

Human-induced macrophage-like cells (hiMacs) were generated from iPSCs (IP-001-1V, XCell Science, Novato, CA, USA) according to a published method described by Takata et al. [34]. In short, 5 ng/mL BMP-4 (314-BP-050, R&D Systems, Minneapolis, MN, USA), 50 ng/mL VEGF (293-VE-500, R&D Systems), and 2 µM CHIR99021 (4423, TOCRIS, Bristol, United Kingdom) induced mesoderm specification of human XCL-1 iPSC colonies and hemangioblast-like cell formation [35]. Replacement of CHIR99021 with 20 ng/mL FGF-2 (223-FB-500, R&D Systems), followed by maintenance of 15 ng/mL VEGF and 5 ng/mL FGF-2, induced differentiation into the hematopoietic lineage according to a modified protocol from Grigoriadis et al. [36]. Wnt signalling was inhibited and hematopoietic stem cells were matured by incubation with 50 ng/mL SCF (255-SC-01M), 10 ng/mL FGF-2, 20 ng/mL IL-3 (203-IL-050, R&D Systems), 10 ng/mL IL-6 (206-IL-050, R&D Systems), 10 ng/mL VEGF, and 30 ng/mL DKK-1(5439-DK-500) for 6 days and with 50 ng/mL SCF, 10 ng/mL FGF-2, 20 ng/mL IL-3, and 10 ng/mL IL-6 for 4 days. To promote terminal differentiation of hiMacs, cells were cultured with 50 ng/mL CSF-1 (216-MC-01M, R&D Systems) for 14 days [37,38,39].

### 2.5. Differentiation of hiMacs to hiMicros

After hiMac generation, hiMacs were positively selected by fluorescence-activated cell sorting (FACS, MoFlo Astrios, Beckman Coulter, Villepinte, France) using CD45-BV605 (304042, Biolegend, Paris, France, 1:300), CD11b-BV421 (301324, Biolegend, 1:300), CD14-FITC (982502, Biolegend, 1:300), CD163-APC (333610, Biolegend, 1:300), and CX3CR1-PE (341604, Biolegend, 1:300). FACS-sorted cells were co-cultured with 70–80% confluent three-week-old (day 0 = induction of neural induction) hiNeurons for three weeks. After three weeks of co-culture, cells were used for imaging. To obtain a pure culture of hiMicros for downstream qPCR analysis, FACS-sorted hiMacs were co-cultured for three weeks on 24-well-plates in inserts (353495, Falcon, pore size 0.4 µm) with confluent three-week-old hiNeurons. During co-culture, 50 ng/mL recombinant human CSF-1 (216-MC-010, R & D Systems) and 50 ng/mL human IL-34 (5265-IL-010, R & D Systems) were added every third day to the culture medium.

### 2.6. Transfer of Conditioned Medium

Cell lines (SK-N-SH, HMC3, and SVGp12) were seeded in 6-well-plates at a density of 1 × 10^5^ cells/cm^2^. Twenty-four hours after seeding, cells were infected with Tha or Th2P-4M at an MOI of 5. After two hours, the viral suspension was removed, and 1 mL of culture medium was added per well. SK-N-SH cells were seeded into 96-well-plates (655086, Greiner Bio-One, Les Ulis, France) at a density of 1.8 × 10^4^ cells/cm^2^. After 24 h, SK-N-SHs previously seeded in 96-well-plates (655086, Greiner Bio) were infected with Tha-eGFP or Th2P-4M-eGFP at an MOI of 0.5. After two hours the viral suspension was removed and replaced via 100 µL of the supernatant taken from previously infected cells (SK-N-SH, HMC3, and SVGp12, at 24 h post-infection) filtered through a 100 kilodalton membrane (28-9322-58, Dominique Dutscher, Bernolsheim, France). After 24 h, the medium was removed, and cells were treated with 2500 U/mL of IFN-α (I4401-100KU, Sigma-Aldrich). Cells were imaged at 48 h post-infection using the Opera Phenix™ High Content Screening System (Perkin Elmer, Villebon-sur-Yvette, France).

### 2.7. Treatment of hiNeurons with Human Recombinant IL-1β, IL-6, and LIF

Human NSCs were seeded at a density of 1.6 × 10^5^ cells/cm^2^ into 96-well-plates (655086, Greiner Bio) and differentiated to hiNeurons as described above. Twenty-one days after differentiation, hiNeurons were infected with Tha-eGFP or Th2P-4M-eGFP at an MOI of 0.5. Two hours after infection, the viral suspension was removed and replaced via 100 µL of NDM or NDM supplemented with 100 ng/mL of recombinant human IL-1β (200-01B, Peprotech, Neuilly-sur-Seine, France), IL-6 (200-06, Peprotech), or LIF (300-05, Peprotech). Cells were imaged at 48 h post-infection using the Opera Phenix™ High Content Screening System (Perkin Elmer).

### 2.8. Opera Phenix™ High Content Screening Assay

Cell lines were seeded at a density of 1.8 × 10^4^ cells/cm^2^. In the case of co-cultures, different cell lines were seeded in equal quantities within wells. Twenty-four hours after seeding, cells were infected with Tha-eGFP and Th2P-4M-eGFP. Twenty-four hours post-infection, cells were treated with 2500 U/mL of IFN-α (I4401-100KU, Sigma-Aldrich) and incubated for 24 h at 37 °C and 5% CO_2_ prior to fixation. Cells were fixed using 4% PFA (Thermo Scientific, J61984) for 15 min at room temperature, washed with PBS (10010023, Thermo Scientific) and permeabilized using 0.5% triton X-100 (648463, Millipore) for 10 min. Cells were stained with primary and secondary antibodies listed according to the manufacturer’s instructions (Table S1). Apoptotic cells were quantified by the in situ cell death detection kit (12156792910, Roche, Meylan, France) according to the manufacturer’s instructions. Dead cells were detected with the ReadyProbes^®^ Cell Viability Imaging Kit (R37610, Thermo Scientific). Images were acquired via the Opera Phenix™ High Content Screening System (Perkin Elmer) using the parameters mentioned in Table S2. To identify eGFP^+^ cells, we used the software Columbus 2.9.1 (Perkin Elmer), which automatically detects nuclei and the cellular cytoplasm. Intensity thresholds to distinguish eGFP^+^ from eGFP^−^ cells were based on the autofluorescence level of non-infected cells (Table S2).

### 2.9. RNA Isolation and cDNA Synthesis

Total RNA was isolated using the RNeasy Mini Kit (74104, Qiagen, Courtaboeuf, France) by the following procedure: EzDNase (11766051, Thermo Scientific) was used to eliminate genomic DNA. Adjacently, 500 ng or 1 μg of purified RNA was converted into first-strand cDNA using the SuperScript™ VILO™ IV enzyme (11756050, Thermo Scientific) according to the manufacturer’s instructions. For real-time qPCR experiments, cDNA was diluted 1/50 or 1/100, respectively.

### 2.10. Quantitative PCR

Quantitative PCR, based on the detection of the SYBR Green dye, was performed by using 2.5 µL of the synthesized and diluted cDNA in the presence of 5 µL QuantiTect SYBR Green (204143, Qiagen) and 1 mM specific primers (Table S3) in a final volume of 10 µL. Oligonucleotides were used for PCR at a concentration of 10 pmol/µL. All the samples were measured in triplicate. Gene expression levels were normalized to the endogenous expression of the housekeeping gene *18S* (Eurofins) and the respective non-infected cells (mock). Quantitative PCR was performed on the 7500 Real-Time PCR System (Thermo Scientific, 7500 Software v2.3) using the following: initial denaturation step (1× repetition, 10 min, 95 °C), amplification step (40 repetitions, 15 s at 95 °C, 1 min at 60 °C), and melting curve determination step (1× repetition, 15 s at 95 °C, 1 min at 60 °C, 15 s at 95 °C, 15 s at 60 °C). Gene expression was normalized to expression of housekeeping gene *18S* (ΔCT), and the difference in gene expression was calculated as the difference between infected and non-infected samples (ΔΔCT) [40].

### 2.11. Protein Quantification

Cells were seeded at a density of 1 × 10^5^ cells/cm^2^ and infected with Tha or Th2P-4M at an MOI of 5. Forty-eight hours post-infection, cells were lysed using Procartaplex™ cell lysis buffer (EPX-99999-000, Thermo Scientific) according to the manufacturer’s instructions to quantify protein expression in the cellular cytoplasm. Intracellular protein concentrations of human IFN-β, IFN-γ, IL-1β, IL-6, IL-15, CXCL10, LIF, CCL5, and TNF-α were quantified using a 9-plex Procartaplex assay (PPX-09, Thermo Scientific). In short, DropArray 96-well-plates (96-CC-BD-05, Clinisciences, Nanterre, France) were blocked using 1% BSA for 30 min. After blocking, 20 µL of cell lysate was added per well. According to the protocol, plates were stepwise incubated with 5 µL detection antibody, 10 µL streptavidin-PE, and 10 µL reading buffer per well before being read by the Luminex 200™ instrument (Thermo Scientific).

### 2.12. Statistical Analysis

Percentages and means ± standard deviation (SD) were calculated with Prism version 9 (GraphPad, San Diego, CA, USA) and R version 4.0.4 (R Foundation, Vienna, Austria). Standard deviation and statistical significance were only calculated when three independent experiments were conducted. Multiple comparisons of data were performed using either Prism version 9 or the lme4 package within R version 4.1.1 (R Foundation, Vienna, Austria), with the test being indicated in the figure legend. If multiple comparisons were performed, significance thresholds were lowered to account for multiple testing. Principal component analysis and hierarchical clustering on gene expression values were performed using R (version 4.0.4). Heatmaps represent normalized gene expression values that were clustered after centring gene expression values on their mean. Figures were generated using Illustrator CC 2019 (Adobe, San Jose, CA, USA).

The analysis of the proportion of eGFP^+^ cells in co-cultures (mixed in equal ratios) performed in Figure S3 was based on the comparison of confidence intervals in each co-culture to a linear combination (with equal weights because the mixture ratios are the same) of the corresponding monocultures. More precisely, the first step of this analysis is the computation of the expected confidence intervals (from monocultures) on the percentages of eGFP^+^ cells for co-cultures (1) and triple cultures (2).

CI_95_ (expected) = 0.5 × CI_95_ (cell type A) + 0.5 × CI_95_ (cell type B) (1)

CI_95_ (expected) = 0.33 × CI_95_ (cell type A) + 0.33 × CI_95_ (cell type B) + 0.33 × CI_95_ (cell type C) (2)

The second step is to compute an observed confidence interval on the actual values measured in the co-cultures. If the observed confidence intervals overlapped with the expected confidence intervals, we concluded that the proportion of eGFP^+^ cells in co-cultures was not a consequence of an interaction between culture cell types. 

## 3. Results

### 3.1. Astrocytic and Microglial Cell Lines Show a Lower Susceptibility to Tha and Th2P-4M Infection Compared to SK-N-SH

**Figure 1:**
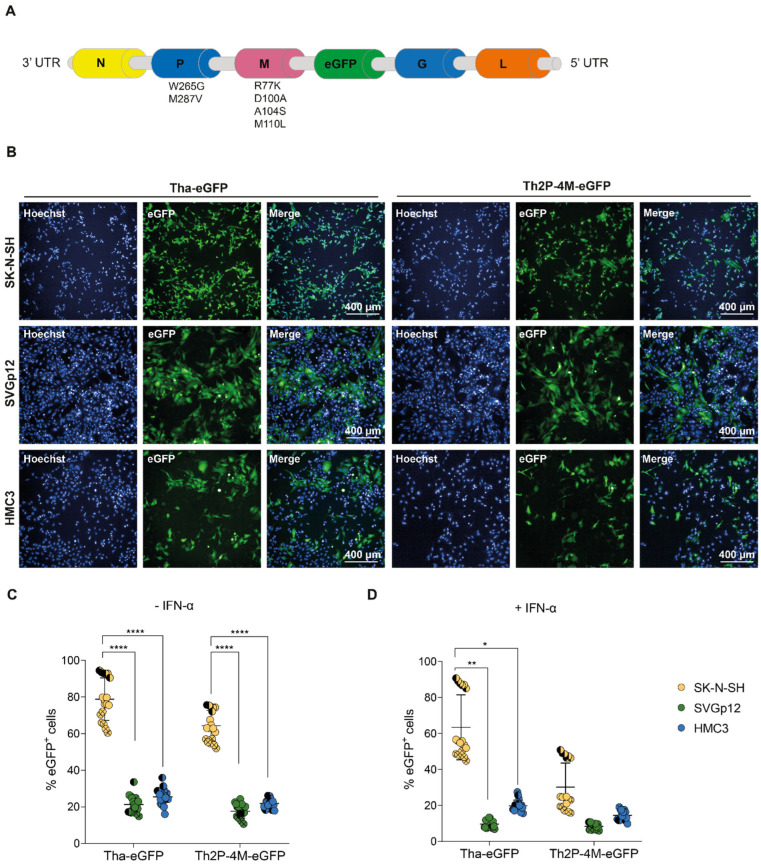
Tha-eGFP and Th2P-4M-eGFP preferentially replicate and spread in human SK-N-SH compared to astrocyte-like (SVGp12) and microglia-like (HMC3) cell lines in vitro. (**A**) Schematic overview of the genomic organization of Tha-eGFP and Th2P-4M-eGFP. Tha-eGFP and Th2P-4M-eGFP harbour the same genetic background except for two mutations introduced in viral P-protein (W265G, M287V) and four mutations introduced in the M-protein (R77K, D100A, A104S, and M110L) [26,31,32]. For imaging purposes, the eGFP sequence was introduced after the M-protein gene sequence as described previously [33]. (**B**) Representative immunofluorescence pictures of SK-N-SH, SVGp12, and HMC3 upon infection with Tha-eGFP and Th2P-4M-eGFP at 48 h post-infection. (**C**) Quantification of eGFP^+^ cells in human CNS monocultures upon Tha-eGFP or Th2P-4M-eGFP infection. (**D**) Quantification of eGFP^+^ cells in human CNS monocultures upon Tha-eGFP or Th2P-4M-eGFP infection and subsequent IFN-α treatment at 24 h post-infection. (**B**–**D**) Cells were infected with Tha-eGFP or Th2P-4M-eGFP (MOI 0.5) and imaged at 48 h post-infection. All experiments were performed three times (*n* = 3) independently. (**C**,**D**) Each dot represents imaging of one well of a 96-well-plate (approx. 8 × 10^3^ cells/well). Bars show mean ± SD. Different colours present different cell types, and different symbol shapes indicate the three technical replicates performed. Hoechst binds to regions of DNA in the minor groove visualizing cellular DNA. The percentages of eGFP^+^ cells were analysed using a mixed model with the replication factor as a random effect, followed by multiple comparisons corrected by Tukey’s method (**** adjusted *p*-value < 0.000016, ** adjusted *p*-value < 0.0016, * adjusted *p*-value < 0.0083). If no *p*-value is indicated, no significant difference was observed. eGFP = Enhanced Green Fluorescent Protein.

Using reverse genetics, we introduced six mutations in conserved residues of the lyssavirus P-protein [32,41] and M-protein [31,42,43] of pathogenic dog RABV Tha to diminish RABV pathogenicity (Figure 1A), as described previously [26]. Upon infection with the two fluorescent RABV strains, Tha-eGFP and Th2P-4M-eGFP, eGFP was expressed in all different CNS cell lines, indicating successful infection of the neuroblastoma cell line SK-N-SH, astrocyte-like SVGp12, and microglia-like HMC3 cells in vitro (Figure 1B).

Quantification of eGFP expression via fluorescence microscopy at 48h post-infection revealed that, on average, 78.9% of SK-N-SH expressed eGFP, whereas significantly less astrocyte-like SVGp12 (21.3%) and microglial-like HMC3 (25.4%) expressed eGFP upon Tha-eGFP infection at an MOI of 0.5 (adjusted *p*-value < 0.000016, Figure 1C). Similarly, Th2P-4M-eGFP induced eGFP expression in significantly less astrocyte-like SVGp12 (17.6%) and microglial-like HMC3 (21.8%) compared to infected SK-N-SH (64.3%, adjusted *p*-value < 0.000016, Figure 1C). Comparing the proportions of infected cells between Tha-eGFP and Th2P-4M-eGFP did not reveal a significant difference between viral strains. Further, viral growth kinetics of Tha and Th2P-4M were assessed in SK-N-SH, SVGp12, and HMC3. No difference was observed between distinct CNS cell types during Tha or Th2P-4M infection at 12, 24, or 36 h post-infection. However, SK-N-SH produced significantly more infectious progeny particles compared to SVGp12 and HMC3 at 48 h post-infection (Figure S1), corroborating the results obtained by the quantification of eGFP expression (Figure 1). Taken together, our results indicate that Tha-eGFP and Th2P-4M-eGFP exhibited a strong neuron-specific tropism in CNS cell lines (Figure 1C).

Adding exogenous IFN-α artificially activates the JAK-STAT pathway, resulting in the expression of ISGs and antiviral proteins. After IFN-α treatment, as expected, Tha and Th2P-4M-eGFP both exhibited a decreased tropism for SK-N-SH cells, although a statistical difference from non-treated SK-N-SH was only observed during Th2P-4M-eGFP infection (adjusted *p*-value < 0.0083) and not during Tha-eGFP infection (adjusted *p*-value > 0.0083, Figure S2). In detail, exogenous IFN-α lowered Tha-eGFP infection of SK-N-SH from 78.9 to 63.5%, and Th2P-4M-eGFP infection was reduced from 64.3 to 30.2% (adjusted *p*-value < 0.0083, Figure S2). The same effect was observed for IFN-α-treated SVGp12 infected with Tha-eGFP (from 21.3 to 9.7%, adjusted *p*-value > 0.0083) and Th2P-4M-eGFP (from 17.6 to 8.4%, adjusted *p*-value < 0.0083, Figure S2). In contrast, the percentage of eGFP^+^ HMC3 decreased only slightly during Tha-eGFP (25.4 to 19.8%, adjusted *p*-value > 0.0083) and Th2P-4M-eGFP infection (from 21.8 to 14.5%, adjusted *p*-value > 0.0083) upon IFN-α treatment compared to non-treated HMC3 (Figure S2). Thus, wild-type viral P- and M-proteins seem to be crucial for evading the IFN-α-induced antiviral responses of neuron- and astrocyte-like cell lines but less important in the case of the microglial cell line HMC3.

### 3.2. RABV Infects Human NSC-Derived hiNeurons and hiAstrocytes but Fails to Infect hiMicros In Vitro

**Figure 2:**
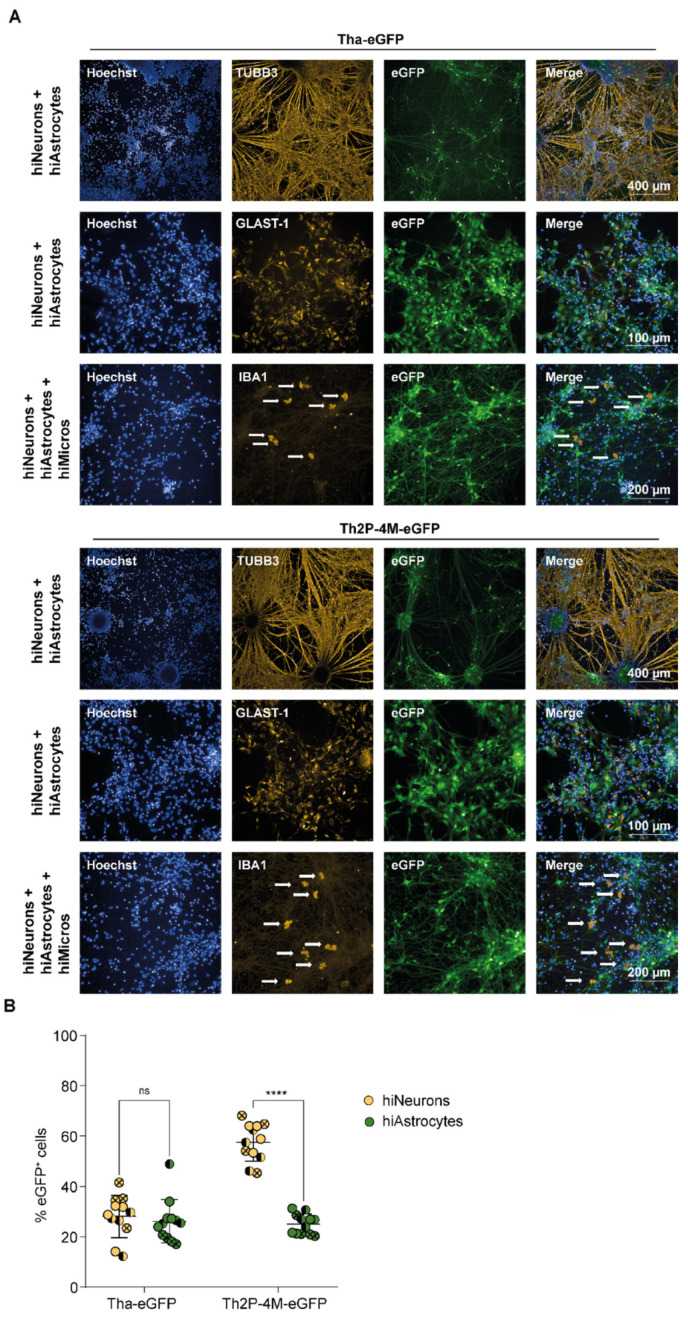
Whereas hiNeurons and hiAstrocytes are highly susceptible to Tha-eGFP and Th2P-4M-eGFP infection, hiMicros are resistant to Tha-eGFP or Th2P-4M-eGFP infection in vitro. (**A**) Representative immunofluorescence pictures of hiNeurons, hiAstrocytes, and hiMicros upon infection with Tha-eGFP and Th2P-4M-eGFP. IBA1^+^ hiMicros are indicated with an arrow. (**B**) Quantification of eGFP^+^ cells in Tha-eGFP-infected triple cultures consisting of hiNeurons, hiAstrocytes, and hiMicros. Every dot represents imaging of one well of a 96-well-plate (approx. 1 × 10^4^ cells/well). Bars show mean ± SD. Different colours present different cell types, and different symbol shapes indicate the three technical replicates performed. Hoechst binds to regions of DNA in the minor groove visualizing cellular DNA. Percentages of eGFP^+^ cells were analysed using a mixed model with the replication factor as a random effect, followed by multiple comparisons corrected by Tukey’s method (**** adjusted *p*-value < 0.000025, ns = no significant difference was observed). (**A**,**B**) Prior to infection, cultures consisted of hiNeurons (71.0%), hiAstrocytes (18.4%), and hiMicros (10.6%, Table S5). Cells were infected with Tha-eGFP or Th2P-4M-eGFP (MOI 0.5) and imaged at 48 h post-infection. All experiments were performed three times (*n* = 3) independently. eGFP = Enhanced Green Fluorescent Protein; GLAST-1 = Glutamate Aspartate Transporter-1; IBA1 = Ionized calcium-binding adaptor molecule 1; TUBB3 = Class III Beta-Tubulin.

After investigating the susceptibilities of immortalized CNS cell lines towards RABV infection, we investigated the role of more relevant CNS cells, particularly the susceptibility of human stem cell-derived CNS cell types towards RABV infection. First, we differentiated hNSC into hiNeurons and hiAstrocytes and iPSC into hiMicros according to a published protocol [34]. After differentiation, cellular fate was validated by immunofluorescence, qPCR, and FACS (Figure 2A, Figures S3 and S4). As commonly observed in differentiation protocols, differentiation of hNSC did not result in a pure monoculture but resulted in a culture containing in average 85.6% hiNeurons (CI_95_ 84.3–86%, Table S4) but also non-negligible proportions of hiAstrocytes (CI_95_ 14–15.7%, Table S4). To obtain hiMicros, we differentiated iPSCs for 25 days to hiMacs as described previously [34]. Subsequently, iPSC-derived hiMacs were co-cultured for three consecutive weeks with hiNeurons and hiAstrocytes to obtain hiMicros [34]. Therefore, our experimental set-up did not allow us to fully reproduce previously obtained results from CNS cell line monocultures (Figure 1) in a stem cell-derived CNS model since we were unable to obtain pure monocultures of primary CNS cell types. 

Prior to infection, cultures revealed on average 71.0% hiNeurons, 18.4% hiAstrocytes, and 10.6% hiMicros (Table S5). Upon infection, Tha-eGFP and Th2P-4M-eGFP successfully replicated in Class III Beta-Tubulin-positive (TUBB3^+^) hiNeurons and Glutamate Aspartate Transporter 1-positive (GLAST-1^+^) hiAstrocytes (Figure 2A). Surprisingly, Tha-eGFP and Th2P-4M-eGFP failed to infect ionized calcium-binding adaptor molecule-positive (IBA1^+^) hiMicros (Figure 2A and Figure S4), suggesting that hiMicros are even more restrictive in their ability to replicate RABV compared to the microglial cell line HMC3 (Figure 1).

Further, Tha-eGFP showed similar rates of infection in hiNeurons (CI_95_: 23–33%) and hiAstrocytes (CI_95_: 21–32%) in triple cultures consisting of hiNeurons, hiAstrocytes, and hiMicros (Figure 2B). Likewise, Th2P-4M-eGFP revealed a similar rate of infection as Tha-eGFP in hiAstrocytes (CI_95_: 22–28%) but exhibited a higher rate of infection in hiNeurons (CI_95_: 53–62%) compared to Tha-eGFP (CI_95_: 23–33%, Figure 2B). Altogether, this demonstrates that hiNeurons and hiAstrocytes are highly susceptible, whereas hiMicros are not susceptible towards Tha-eGFP and Th2P-4M-eGFP infection (Figure 2 and Figure S4).

### 3.3. Tha Induces Only Modest Innate Immune Responses in Cells of Neuronal Origin Whereas Glial Cells Strongly Respond to Tha Infection

**Figure 3:**
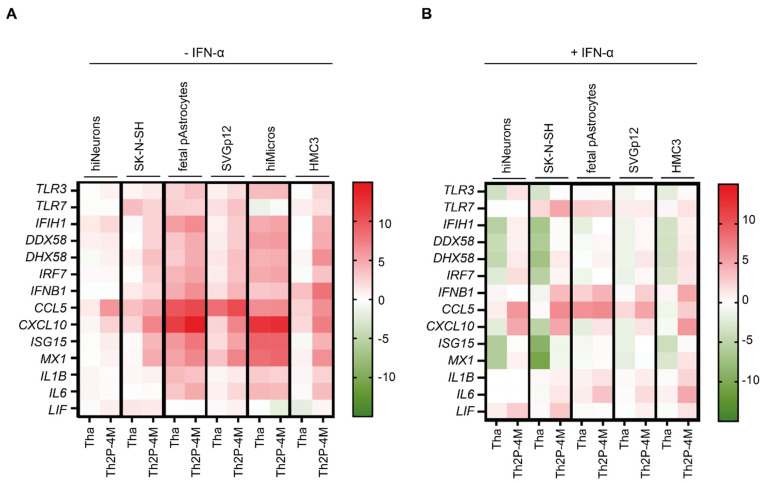
Tha is highly adapted to evade the neuronal innate immune system. (**A**) Expression of innate immune genes upon Tha and Th2P-4M infection. (**B**) Expression of innate immune genes upon Tha and Th2P-4M infection with subsequent IFN-α treatment at 24 h post-infection. (**A**,**B**) Cells were infected with Tha or Th2P-4M (MOI 5), and gene expression was quantified at 48 h post-infection via qPCR. To obtain hiNeurons, hNSC were differentiated for 21 days, resulting in cultures that contained 85.6% hiNeurons (Table S4). hiMacs were differentiated to hiMicros by co-culturing them for three consecutive weeks in inserts with hiNeurons (see Section 2). All experiments were performed three times (*n* = 3) independently. Heatmaps present gene expression (ΔΔCT), which was normalized to the expression of the reference gene *18S* and the respective mock-infected (**A**) or IFN-α-treated mock (**B**). Colour scaling presents differences observed in gene expression (ΔΔCT) between infected and respective non-infected controls.

To further characterize the susceptibilities of CNS cell types towards RABV infection, CNS cell types were cultured separately, and the expression of a panel of selected innate immunity genes (*TLR3*, *TLR7*, *IFIH1*, *DDX58*, *DHX58*, *IRF7*, *IFNB1*, *CCL5*, *CXCL10*, *ISG15*, *MX1*, *IL1B*, *IL6*, and *LIF*) that are known to be involved in the immune response towards RABV infection was quantified via qPCR (Figure 3 and Figure S5). For this experiment, we used the CNS cell lines SK-N-SH, SVGp12, and HMC3 (Figure 1), hNSC-derived hiNeurons (Figure 2A), commercially purchased foetal pAstrocytes, and iPSC-derived hiMicros which were obtained by co-culturing hiMacs in cell culture inserts with hNSC-derived hiNeurons. Although these experiments were performed both in human cell lines and in differentiated human CNS cells, here we will focus our attention on the results obtained from differentiated human CNS cells.

Comparing basal expression levels of innate immunity genes between CNS cell types by qPCR revealed that hiMicros present a strong basal expression of innate immune receptors (*IFIH1*, *DDX58*, *DHX58*, and *TLR3*), the adaptor molecule *IRF7*, chemokine *CCL5,* and the antiviral protein *MX1 (*Figure S5A). In contrast, foetal pAstrocytes revealed a strong expression of the RLR *DDX58* (encoding RIG-I), and SK-N-SH showed a strong expression of *TLR3*, although this was not observed in hiNeurons (Figure S5A). Upon IFN-α treatment, foetal pAstrocytes strongly amplified the inflammatory response via the expression of *TLR3*, *IFIH1*, *DDX58*, *DHX58*, *IRF7*, *ISG15*, and *MX1* (Figure S5B). In contrast, hiNeurons showed little response to IFN-α stimulation (Figure S5B). In detail, *DDX58*, *ISG15*, and *MX1* were the only genes that were modestly modulated by IFN-α in hiNeurons (Figure S5B). Additionally, we also noticed a strong and specific amplification of *CXCL10* in foetal pAstrocytes upon IFN-α treatment (Figure S5B).

**Figure 4:**
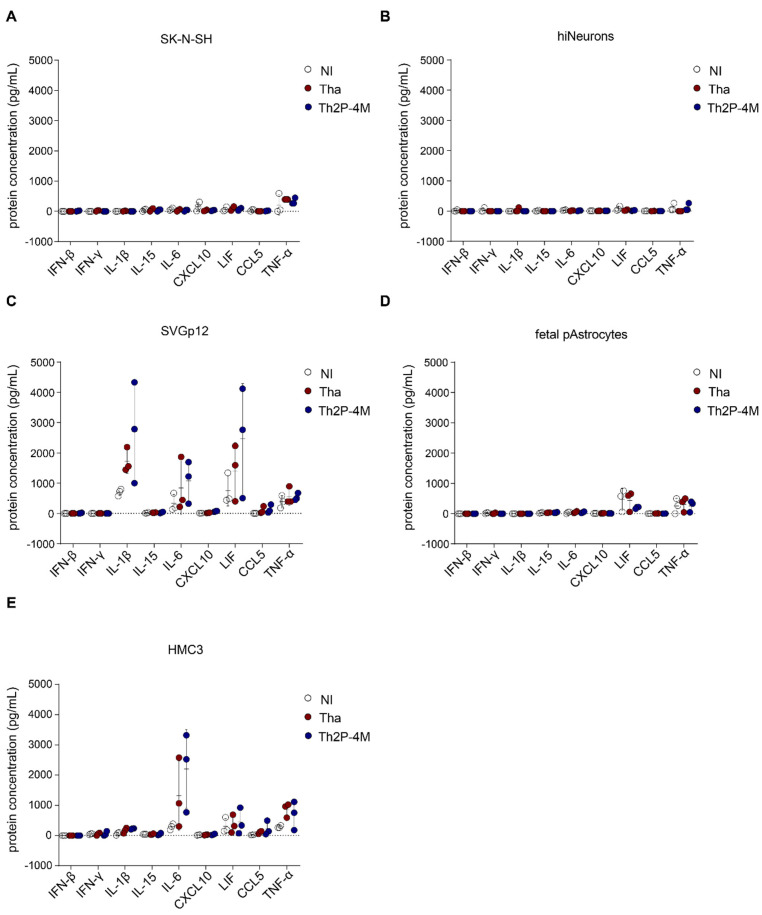
Modulation of intracellular protein expression (IL-1β, IL-6, and LIF) in Tha- and Th2P-4M-infected glial cells. (**A**) Intracellular protein concentrations of SK-N-SH cells upon Tha or Th2P-4M infection. (**B**) Intracellular protein concentrations of hiNeurons upon Tha or Th2P-4M infection. (**C**) Intracellular protein concentrations of astrocyte-like SVGp12 cells upon Tha or Th2P-4M infection. (**D**) Intracellular protein concentrations of foetal pAstrocytes upon Tha or Th2P-4M infection. (**E**) Intracellular protein concentrations of microglia-like HMC3 cells upon Tha or Th2P-4M infection. (**A**–**E**) Cells were infected with Tha or Th2P-4M (MOI 5), and intracellular protein concentration was quantified at 48 h post-infection using the DropArray system (Curiox). All experiments were performed three times (*n* = 3) independently. All bars show mean ± SD with a one-way ANOVA analysis. To correct for multiple testing, the *p*-value was corrected accordingly. If no *p*-value is indicated, no significant difference was observed.

In the next step, we quantified the expression of innate immunity genes upon Tha and Th2P-4M infection at 48 h post-infection via qPCR (Figure 3A or Figure 4B). We deliberately used a high level of MOI (MOI 5) to ensure that all cells have been in contact with infectious viral particles. First, Th2P-4M induced a stronger innate immune response compared to Tha. Foetal pAstrocytes and hiMicros strongly induced the expression of innate immunity genes upon Tha and Th2P-4M infection, which was less observed in respective astrocyte-like (SVGp12) and microglia-like (HMC3) cell lines. Further, few differences were observed among CNS cell lines (SK-N-SH, SVGp12, and HMC3), whereas clear differences were observed in the innate immune response among hiNeurons, foetal pAstrocytes, and hiMicros (Figures S6 and S7). This further illustrates the differences between primary CNS cells and cell lines. In the following section, we base our conclusions on the most-relevant model, namely primary CNS cell types. Upon Tha and Th2P-4M infection, foetal pAstrocytes strongly induced the expression of *IFIH1*, *CCL5*, and *CXCL10*, whereas hiMicros strongly upregulated expression of *CXCL10* and genes coding for antiviral proteins *ISG15* and *MX1* (Figure 3A).

Subsequently, we stimulated cells with IFN-α at 24 h post-infection to artificially activate the JAK-STAT pathway. Expression of innate immunity genes was quantified at 48 h post-infection to examine if Tha or Th2P-4M can inhibit the IFN-α-induced inflammatory response (Figure 3B). First, we assessed expression of human *IFNAR1* and *IFNAR2* (Figure S8) to better understand the distinct cellular responses towards IFN-α treatment. Overall, *IFNAR1* expression was higher than *IFNAR2*, disregarding the CNS cell type investigated. Further, *IFNAR1* and *IFNAR2* expression exhibited a gradient, with higher expression observed in hiMicros followed by foetal pAstrocytes and hiNeurons (Figure S8).

Regarding the expression of ISGs, clear differences were observed between cells lacking IFN-α treatment (Figure 3A). Strikingly, cell type-specific differences were less emphasized after IFN-α stimulation (Figure 3B, Figures S6 and S7) despite the strong differences in *IFNAR2* expression among the distinct CNS cell types (Figure S8). Despite IFN-α treatment, virulent Tha suppressed the expression of innate immunity genes compared to less virulent Th2P-4M in all CNS cell types. However, differences in innate immune gene expression between Tha- and Th2P-4M-infected cells were more evident in SK-N-SH and hiNeurons compared to glial cells (Figure 3A). In contrast to Th2P-4M, Tha strongly downregulated the expression of *TLR3*, RLRs (*IFIH1*, *DDX58*, and *DHX58*), and the antiviral proteins *MX1* and *ISG15* in IFN-α-treated cells of neuronal origin (SK-N-SH and hiNeurons) as well as expression of RLRs (*IFIH1*, *DDX58*, and *DHX58*) and *ISG15* in microglia-like HMC3. Further, IFN-α-treated foetal pAstrocytes strongly expressed *TLR7*, *IFNB1*, *CCL5*, *IL1B*, and *IL6* upon Tha infection. IFN-α-treated microglia-like HMC3 upregulated *IFNB1*, *CCL5*, *CXCL10*, and *IL6* upon Tha infection (Figure 3B).

Overall, we show that Tha induced few innate immune responses in hiNeurons and even in some cases suppressed the expression of innate immunity genes compared to Th2P-4M, whereas glial cells (foetal pAstrocytes and hiMicros) induced a strong innate immune response upon Tha and Th2P-4M infection (Figure 3, Figures S6 and S7).

In a further step, we quantified intracellular proteins corresponding to genes that were identified in our previous transcriptomic analysis (IFN-β, IL-1β, IL-6, CXCL10, LIF, and CCL5; Figure 3), and a few additional proteins of interest (IFN-γ, IL-15, and TNF-α; Figure 4). Intracellular protein concentrations revealed the induction of cell type-specific immune responses upon RABV infection: both neuronal cell types investigated, SK-N-SH and hiNeurons, did not express any of the aforementioned proteins upon Tha or Th2P-4M infection but constitutively expressed modest levels of TNF-α (Figure 4A,B). In glial cells, Tha and Th2P-4M infection did not induce significant expression of the selected proteins, although modest modulations of constitutively expressed proteins (IL-1β, IL-6, LIF) that were detected in astrocytic cells and/or in microglia-like HMC3 (Figure 4C–E) were recorded.

Subsequently, we investigated if some glial-expressed cytokines can restrict RABV replication in hiNeurons or hiAstrocytes via the induction of secondary signalling pathways. Therefore, hiNeurons and hiAstrocytes were infected with Tha-eGFP or Th2P-4M-eGFP and treated with human recombinant IL-1β, IL-6, or LIF two hours after infection. Forty-eight hours post-infection, the proportion of eGFP^+^ hiNeurons and hiAstrocytes was quantified by fluorescence microscopy (Figure S9). Fluorescence imaging revealed that neither IL-1β (Figure S9A,B), IL-6 (Figure S9C,D), nor LIF (Figure S9E,F) significantly modulated the percentage of eGFP^+^ hiNeurons or hiAstrocytes at 48 h post-infection. Further, neither IL-1β, IL-6, nor LIF lowered the percentage of dead cells in Tha-eGFP-infected or Th2P-4M-infected hNSC-derived CNS cultures (Figure S10).

### 3.4. Glial Cell Lines Constitutively Protect SK-N-SH from Tha Infection

**Figure 5:**
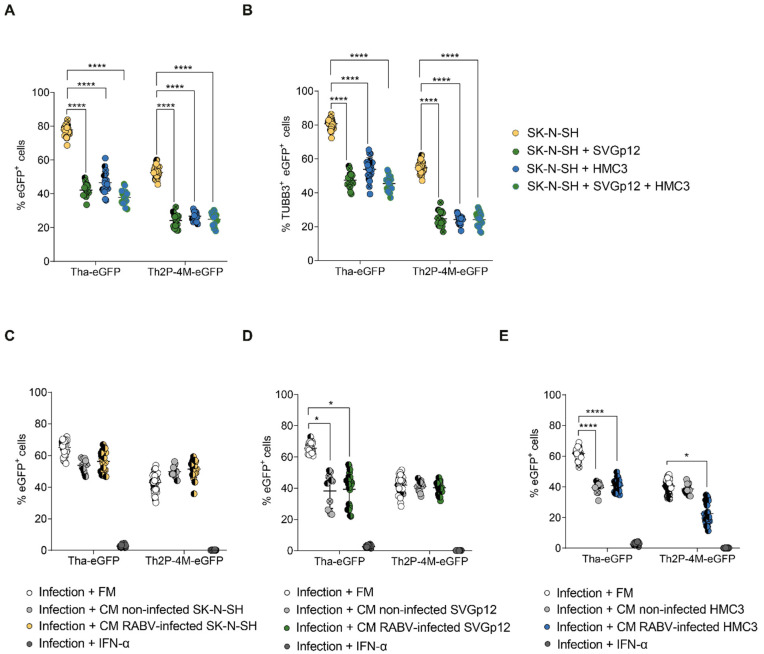
Astrocyte-like SVGp12 and microglia-like HMC3 constitutively protect SK-N-SH from Tha-eGFP infection in vitro. (**A**) Quantification of eGFP^+^ cells in mono- and co-cultures. (**B**) Quantification of TUBB3^+^ eGFP^+^ cells in mono- and co-cultures. (**A**,**B**) Mono- or co-cultures were infected with Tha-eGFP or Th2P-4M-eGFP (MOI 0.5) and imaged at 48 h post-infection using the Opera Phenix^®^ High Content Screening System (Perkin Elmer). (**C**–**E**) Quantification of eGFP^+^ cells in SK-N-SH monocultures. After infection with Tha-eGFP or Th2P-4M-eGFP (MOI 0.5), SK-N-SH cells were incubated with the filtered fresh medium (FM) or filtered conditioned medium (CM) from non-infected or homotypic infected (MOI 5, 24 h post-infection) SK-N-SH (**C**), SVGp12 (**D**), or HMC3 (**E**). In detail, Tha-eGFP-infected cells were only treated with supernatants from cells previously infected with Tha-eGFP. Correspondingly, Th2P-4M-eGFP-infected cells were only treated with supernatants from cells previously infected with Th2P-4M-eGFP. Cells were imaged at 48 h post-infection. (**A**–**E**) All experiments were performed three times (*n* = 3) independently. Each dot represents imaging of one well of a 96-well-plate (approx. 8 × 10^3^ cells/well). Bars show mean ± SD. Different colours present different biological conditions, and different symbol shapes indicate the three technical replicates performed. The percentages of eGFP^+^ cells or eGFP^+^ TUBB3^+^ cells were analysed using a mixed model with the replication factor as a random effect, followed by multiple comparisons corrected by Tukey’s method (**** adjusted *p*-value < 0.000016, * adjusted *p*-value < 0.0083). (**C**–**E**) Comparisons between all conditions and infection + IFN-α treatment were significant but not indicated in the figure. For the remaining comparisons, if no *p*-value is indicated, no significant difference was observed.

Since we were unable to identify protective abilities of either IL-1β, IL-6, or LIF during RABV infection, we aimed to characterize the interplay of CNS cell types during RABV infection using co-cultures of CNS cell types. As we were unable to obtain pure stem cell-derived CNS monocultures, these studies were performed on the different CNS cell lines described previously (Figure 1). More specifically, we assessed whether glial cells limit RABV infection in RABV-infected CNS cell line co-culture models. Therefore, we compared the percentage of infection of SK-N-SH monocultures to co-cultures of SK-N-SH and astrocyte-like cells (SK-N-SH + SVGp12), co-cultures of SK-N-SH and microglia-like cells (SK-N-SH + HMC3), and co-cultures of SK-N-SH, astrocyte- and microglia-like cells (SK-N-SH + SVGp12 + HMC3, Figure 5A). Although we are fully aware that the ratio of neuronal cells may vary between different brain regions, cells were mixed in equal ratios (1:1 for co-cultures, 1:1:1 for triple cultures) for practical purposes. During Tha-eGFP infection, the presence of SVGp12 (42.2%), HMC3 (46.6%), or both cell types (37.9%) significantly reduced the proportion of total eGFP^+^ cells (adjusted *p*-value < 0.000016) compared to SK-N-SH monocultures (77.8%). Similarly, culturing SVGp12 (24.2%), HMC3 (26.8%), or both cell types (24.9%) in co-cultures with SK-N-SH significantly reduced the proportion of total eGFP^+^ cells (adjusted *p*-value < 0.000016) compared to SK-N-SH monocultures (52.6%) during Th2P-4M-eGFP infection (Figure 5A). However, comparing the observed percentage of total eGFP^+^ cells (Figure 5A) with the percentage we would have expected from lower susceptibilities of glial cells (Figure 1) revealed only a significant reduction of the percentage of total eGFP^+^ cells during Th2P-4M-eGFP infection but no significant reduction during Tha-eGFP infection (Figure S11), suggesting that Th2P-4M-eGFP induces a cellular crosstalk that exhibits protective capacities on CNS cultures. To further investigate the effect of co-cultures on the percentage of infected SK-N-SH, the proportion of TUBB3^+^ (Supplementary Table S1) eGFP^+^ cells was determined, which corresponds to the proportion of infected SK-N-SH (Figure 5B). Cells of glial origin, SVGp12, HMC3, or SVGp12 and HMC3 together, significantly reduced the percentage of eGFP^+^ TUBB3^+^ SK-N-SH during both Tha-eGFP (adjusted *p*-value < 0.000016, Figure 5B) and Th2P-4M-eGFP infection (adjusted *p*-value < 0.000016, Figure 5B). In short, the addition of any glial cell type to SK-N-SH cells reduced the percentage of infected SK-N-SH cells disregarding the viral strain used. The addition of a third cell line (adding SVGp12 and HMC3 simultaneously) did not change this reduction of percentage, suggesting that the observed effect is not simply the result of viral particles binding to the additional cell types but rather due to a resistance mediated by either astrocyte-like (SVGp12) or microglia-like (HMC3) cells to neuron-like cells (SK-N-SH) during RABV infection.

Further, to confirm that the effect observed resulted from the interplay between different neuronal cell lines, we investigated if glial-mediated restriction of SK-N-SH infection is mediated via the secretion of glial-derived proteins to the supernatant. Therefore, we transferred the medium of non-infected or homotypic infected SK-N-SH, SVGp12, or HMC3 cells after viral particle removal to freshly infected SK-N-SH cells to assess if the conditioned medium reduces the percentage of eGFP^+^ SK-N-SH compared to the transfer of fresh cell culture medium (Figure 5C–E). Exogenous IFN-α was used as a positive control since it was previously shown (Figure 1C,D) to significantly restrict viral infection. Restriction of RABV infection by IFN-α was confirmed (Figure 5C–E, adjusted *p*-value < 0.0016). As expected, transfer of conditioned medium originating from non-infected or RABV-infected (independent of the viral strain used) SK-N-SH to respective RABV-infected SK-N-SH cells did not significantly lower the percentage of RABV-eGFP^+^ SK-N-SH (Figure 5C). This suggests that in our experimental set-up, there is no crosstalk that can be measured in our assay between RABV-infected SK-N-SH. In contrast, we observed a constitutive effect of SVGp12 and HMC3 independent of homotypic infection: transfer of non-infected or Tha-eGFP-infected SVGp12 (adjusted *p*-value < 0.0083, Figure 5D) or HMC3 (adjusted *p*-value < 0.000016, Figure 5E) significantly reduced the percentage of eGFP^+^ SK-N-SH in Tha-eGFP infected cultures. Overall, and in contrast to SK-N-SH, non-infected and Tha-infected SVGp12 and HMC3 constitutively protect SK-N-SH from Tha-eGFP infection via the secretion of glial-derived proteins (Figure 2C–E).

These constitutive effects, however, were not observed during Th2P-4M-eGFP infection: transfer of supernatant originating from non-infected or Th2P-4M-eGFP-infected SVGp12 (Figure 5D) or from non-infected HMC3 (Figure 5E) had no effect on the proportion of eGFP^+^ SK-N-SH. Only the transfer of supernatant originating from Th2P-4M-eGFP-infected HMC3 significantly reduced the proportion of Th2P-4M-eGFP^+^ SK-N-SH (adjusted *p*-value < 0.0083, Figure 5E). This means that the reduction of eGFP^+^ TUBB3^+^ cells by adding SVGp12 to the cultures (Figure 5B) is not mediated by transferring the supernatant of SVGp12 onto Th2P-4M-infected SK-N-SH (Figure 5D). On the other hand, the reduction of eGFP^+^ TUBB3^+^ cells by adding HMC3 to the cultures (Figure 5B) is mediated by transferring the supernatant of HMC3 onto Th2P-4M-infected SK-N-SH (Figure 5E).

## 4. Discussion

In this section, we describe different factors that determine RABV tropism. The first factor relies on the divergent susceptibilities of CNS cell types towards Tha infection. We report that the two RABV strains (Tha-eGFP and its attenuated variant Tha2P-4M-eGFP) used in this study infect hiNeurons and hiAstrocytes, whereas hiMicros are not susceptible to RABV infection in vitro (Figure 3A and Figure S4). Although human CNS cell lines did not fully corroborate these results (Figure 1), Tha-eGFP and Th2P-4M-eGFP still displayed a higher neuron-specific tropism in CNS cell lines (Figure 1C,D). The main difference, however, is the low susceptibility of microglial cell line HMC3 (Figure 1) compared to the absence of RABV infection recorded in iPSC-derived human microglia (Figure 2 and Figure S4). In short, our results question the ability of canine RABV strains to successfully enter and replicate in human microglia (Figure 2). Previously, Ray and colleagues reported that the tissue culture-adapted ERA strain and mouse-adapted bat SRV strain successfully replicate in primary adult human microglia in vitro [19]. Despite these dissimilarities, we suggest that the different nature of RABV strains, their culture adaptation, or insufficient cell purification or differentiation might account for different susceptibilities towards RABV infection observed between these studies. Microscopic analysis of post mortem human brain tissues of rabid patients revealed enhanced activation of microglia surrounding degenerated neurons [44], which are referred to as Babes nodules [45] and observed in other viral encephalitis and infectious disorders [46]. Given that microglia phagocytes degenerate neurons and thereby take up RABV components [44], viral transcripts that are detected in microglia [47] do not necessarily mean that microglia actively support RABV infection. Nevertheless, more research is needed to elucidate the susceptibility and function of microglia in human rabies infection, particularly by using more sophisticated models that reflect the complexity of the human CNS. 

The second factor determining RABV tropism relies on the type and extent of the immune response induced by RABV infection in the different CNS cell types. A limited comparative transcriptomic analysis between Tha- and Th2P-4M-infected cells focusing on innate immune gene expression implied in RABV pathogenesis (Figure 3) revealed that Tha specifically evades neuronal immune responses. Further, we confirm that the evasion of the NF-κB and JAK-STAT pathways is mediated by the specific domains of P- and M-proteins as shown by the transcriptome comparison between Tha and Th2P-4M (Figure 3). Although antiviral signalling via IFNs was originally considered as a universal mechanism to control viral infections, recent evidence suggests that neurons lack robust innate immune signalling pathways to minimize the detrimental effects of viral infection on this non-renewable cell population [48,49]. Further, the neuronal ability to respond to IFN stimulation seems limited given the low gene expression of *IFNAR1* and *IFNAR2* (Figure S8) and the moderate induction of innate immune gene expression following IFN stimulation compared to cells of glial origin (Figure S5B). In contrast to neuronal impairment to respond to Tha infection via IFN induction, we show that glial cells induce strong innate immune responses upon Tha and Th2P-4M infection (Figure 3). Consequently, we suggest that the pronounced neuron-specific tropism of Th2P-4M in cultures consisting of hiNeurons and hiAstrocytes results from its limited or delayed capacity to evade strong immune responses compared to Tha (Figure 2B) [49].

Apart from the crucial role of neurons during RABV infection, our results indicate that astrocytes may also play an important role during infection: in our study, astrocytes strongly induce the transcription of cytokines and adaptor molecules (*IFIH1*, *TLR7*, *IFNB1*, *CCL5*, *CXCL10*, *IL1B*, and *LIF*) upon Tha and Th2P-4M infection in vitro (Figure 3A). We assume that astrocytes sense Tha via RLRs (*IFIH1*, *DDX58*, and *DHX58*) and TLRs (*TLR3* and *TLR7*), in turn inducing the expression of type I IFNs (*IFNB1*), chemokines (*CCL5* and *CXCL10*), and interleukins (*IL1B*). Both IFN-β and IL-1β are known to increase BBB permeability, to activate monocytes, microglia, and astrocytes, and to induce the production of neuroprotective mediators [50]. Previously, astrocytic expression of *CCL5* and *CXCL10* chemokines was shown to induce the recruitment of macrophages, dendritic cells, lymphocytes, and neutrophils, and to regulate microglial activity as well as astrocyte survival [50]. Further, it has been shown that murine astrocytes strongly respond to infection with a recombinant RABV carrying the G-protein of the CVS strain (SAD-G_CVS_) via RLR- and TLRs-induced expression of *IFN-β* in vivo. As a consequence, the IFN response was shown to abort RABV infection of astrocytes in mice [21]. In contrast to attenuated RABV strains, a recent quantitative analysis of RABV tropism in rats revealed a strong tropism for astrocytes by RABV field strains (8–27%). In accordance with these data, we showed that Tha is able to infect human astrocytes in vitro (Figure 2) despite the induction of a strong astrocyte-mediated immune response upon infection (Figure 3). Although human astrocytes constitutively express modest levels of LIF, IL-1β, and IL-6 (Figure 4), none of those factors actively restricted RABV replication in hNSC-derived CNS cultures (Figure S9). Still, the fact that the antiviral activity of LIF [51,52], IL-1β [53], and IL-6 [54,55] has been described previously in different in vitro and in vivo models for various human pathogens urges further research to understand the pleiotropic nature of these interleukins during viral infections, particularly during RABV-mediated encephalitis.

Neurons and astrocytes are not the only cell types playing important roles during RABV infection: here, we identified the upregulation of IFN-inducible genes with antiviral responses (*CXCL10*, *ISG15*, and *MX1*) in hiMicros (Figure 3) as well as a modest increase in IL-6 protein expression in microglial-like cells upon Tha and Th2P-4M infection (Figure 4). Microglia are already known to induce *CXCL10* [56,57] and *ISG15* [47,57,58] expression during viral infection. Generally, activated microglia are well known to release pro-inflammatory cytokines in pathological conditions such as IL-6, resulting in cytotoxicity, immune activation, neuronal excitotoxicity, and apoptosis [59]. Here, we show that IL-6 does not directly restrict RABV replication in either hiNeurons or in hiAstrocytes (Figure S9), nor does it reduce the percentage of dead cells in Tha-eGFP- or Th2P-4M-infected co-cultures (Figure S10). Despite the lack of IL-6-mediated antiviral or anti-apoptotic activity in our model, IL-6 might potentially modulate other biological processes in the CNS, such as microglia and T-cell activation, BBB permeability [60], or synaptic function [61]. 

The third factor determining RABV tropism relies on the interplay between the different CNS cell types. Although we were unable to characterize this factor in differentiated CNS cell types, our results show the importance of the communication between the different CNS cell types during RABV infection. Apart from the simple presence of glial cells in co-cultures, the transfer of supernatant from glial cells protected neuron-like cells from Tha infection (Figure 5). Possible underlying mechanisms comprise glial-mediated signalling directly via cell-to-cell contacts (Figure 5), the induction of inflammatory genes (Figure 3), as well as the expression of cell type-specific inflammatory proteins (Figure 4) that can induce secondary signalling cascades in surrounding neuronal cells. Even though we could not identify the precise factors restricting Tha infection in glial cells (Figure S9), we provide evidence that glial immune responses partly shape RABV tropism. This has already been shown for poliovirus, where the type I IFN system induces the expression of ISGs, particularly PKR and OAS in atrophic tissues [62]. Further, the IFN system dictates viral tropism for VSV [63], West Nile Virus [64], and neurotropic coronavirus [65]. Thus, we urge further research to characterize the interplay between CNS cell types during RABV infection by studying more complex CNS models such as brain organoids or human brain sections. 

## 5. Conclusions

**Figure 6:**
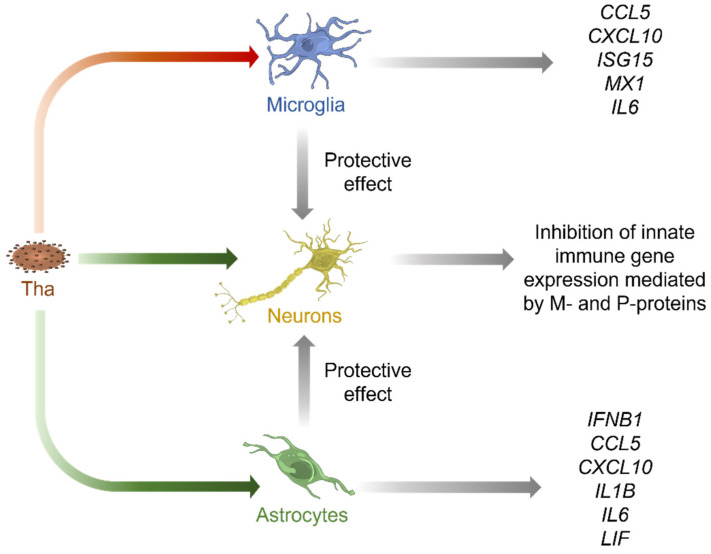
Proposed model of cell type-specific interactions in Tha-infected human cultures consisting of neurons, astrocytes, and microglia. Tha productively infects neurons (yellow) and astrocytes (green), whereas microglia (blue) are not susceptible to Tha infection in vitro (Figure 2). Further, astrocytes and neurons strongly induce expression of innate immune genes upon challenge with Tha virus (Figure 3 and Figure 4). In contrast, P- and M-proteins of Tha inhibit the induction of innate immune genes in neurons (Figure 3). Additionally, we show that the transfer culture medium of Tha-infected astrocytes and microglia protects neurons from Tha infection (Figure 5). Green arrows indicate productive infection while the red arrow indicates abortive infection.

Overall, our results point out that innate immune responses of human CNS cell types towards RABV infection in vitro strongly vary according to the model system used or the CNS cell type investigated (Figure 6).

Whereas neurons lack major antiviral signalling pathways to restrict Tha infection, astrocytes and microglia mount strong immune responses towards Tha infection in vitro. Further, glial cell lines modulate the susceptibility of neurons towards Tha infection. Altogether, this further demonstrates the crucial role of glial cells in limiting RABV infection of neurons. Nevertheless, we need to admit that gene and protein expression levels in CNS cell types were measured in monocultures that do not reflect the complexity of the CNS. Cellular interactions strongly influence immune responses during physiological and pathological states [66], consequently limiting the validity of our study to extrapolate data directly to the human CNS. Thus, more sophisticated models such as suitable human ex vivo CNS models and single-cell approaches are needed to elucidate the underlying cell type-specific differences in mounting innate immune responses upon RABV infection. Apart from the role of glial cells in restricting RABV replication in neurons, we further demonstrate the crucial role of accessory functions of viral P- and M-proteins in viral-mediated immune evasion, which might potentially be mediated via viral interference with NF-κB and JAK-STAT pathways [26,27,31,32,41]. Finally, we conclude that more research is needed to understand the underlying pathways defining RABV tropism and cell type-specific immune responses. The characterization of transcriptome profiles of different CNS cell types would help us understand their intimate interplay during RABV infection and its implications in RABV tropism.

## Data Availability

The data presented in this study are available in additional files supplied by the authors.

## Supplementary Materials

The following supporting information can be downloaded at: https://www.mdpi.com/article/10.3390/v15010088/s1, Figure S1. Comparative kinetics of Tha and Th2P-4M in SK-N-SH, HMC3, and SVGp12 cells; Figure S2. Influence of IFN-α treatment on eGFP expression in Tha-eGFP- and Th2P-eGFP-infected CNS cell lines; Figure S3. Validation of cellular fate by quantification of marker gene expression via RT-qPCR; Figure S4. Detailed image of hiMicros (indicated with an arrow) in Th2P-4M-eGFP-infected cultures (MOI 0.5) 41 consisting of hiNeurons, hhiAstrocytes, and hiMicroglia at 48 h post-infection; Figure S5. Basal expression of innate immunity genes in CNS cell types in vitro. (A) Basal gene expression of a panel 48 of innate immunity genes of different CNS cell types. (B) Basal gene expression of a panel of innate immunity genes 49 of different CNS cell types with subsequent IFN-α stimulation; Figure S6. Innate immune responses of CNS cell types upon Tha and Th2P-4M infection (without IFN-α treatment); Figure S7. Innate immune responses of CNS cell types upon Tha and Th2P-4M infection with subsequent IFN-α 74 treatment; Figure S8. Expression of human interferon receptors (IFNAR1 and IFNAR2) in neurons, astrocytes, and microglia; Figure S9. Neither IL-1β, IL-6, nor LIF protect human hiNeurons or hiAstrocytes from Tha-eGFP or Th2P-4M-eGFP 100 infection in vitro; Figure S10. Neither IL-1β, IL-6 nor LIF significantly modulates the percentage of PI+ hNSC-derived hiNeurons and 120 hiAstrocytes upon Tha-eGFP or Th2P-4M-eGFP infection; Figure S11. Statistical comparison of confidence intervals between observed and expected quantification of eGFP+ cells 128 in co-cultures; Table S1. Antibodies used for fluorescence microscopy; Table S2. Technical parameter used for image acquisition by OPERA Phenix High Content Screening (Perkin Elmer) 147 and subsequent analysis by Columbus Analysis Software (Perkin Elmer); Table S3. Primer sequences used to measure human gene expression via RT-qPCR primers; Table S4. Determination of percentage of hiNeurons (=small nuclei) and hiAstrocytes (=big nuclei) after neural 156 differentiation of human neural stem cells for 21 days; Table S5. Determination of percentage of hiNeurons (=small nuclei), hiAstrocytes (=big nuclei), and hiMicros (IBA1+) 165 in human stem cell-derived CNS cell triple cultures; Table S6. Determination of percentage of hiNeurons (=small nuclei) and hiAstrocytes (=big nuclei) in human NSC- 176 derived CNS cell cultures.

## Abbreviations

CI_95_ = 95% confidence interval; CNS = central nervous system; CNSC = complete neural stem cell; DAMPS = danger-associated molecular patterns; eGFP = enhanced green fluorescent protein; G-protein = glycoprotein; GLAST-1 = Glutamate Aspartate Transporter 1; hiMacs = human induced pluripotent stem cell-derived macrophage-like cells; hiMicros = human induced pluripotent stem cell-derived microglia-like cells; HMC3 = Human Microglia Clone 3; hNSC = human neural stem cells; IBA1 = Ionized calcium-binding adaptor molecule; IFNAR = IFN alpha and beta receptor subunit; IFN-β = interferon beta; IL-1β = interleukin 1 beta; IL-6R = interleukin 6 receptor; iPSC = induced pluripotent stem cells; IRF-3 = interferon regulatory factor 3; ISGs = interferon-stimulated genes; JAK = janus kinase; L-protein = large protein; LIF = leukaemia inhibitory factor; M-protein = matrix protein; mGluR2 = metabotropic glutamate receptor subtype 2; MOI = multiplicity of infection; nAChR = nicotinic acetylcholine receptor; NCAM = neuronal cell adhesion molecule; NDM = neural differentiation medium; NF-κB = nuclear factor kappa-light-chain-enhancer of activated B cells; NOD-like receptors = nucleotide-binding oligomerization domain-like receptors; N-protein = nucleoprotein; PAMPS = pathogen-associated molecular patterns; hiAstrocytes = human induced NSC-derived astrocytes; hiNeurons = human induced NSC-derived neurons; P-protein = phosphoprotein; RABV = rabies virus; RIG-I = retinoic acid-inducible gene I; RLRs = retinoic acid-inducible gene I-like helicases; PRRs = pattern recognition receptors; p75NTR = low-affinity p75 neurotropin receptor; STAT = signal transducer and activator of transcription; SVGp12 = human foetal glial cell line; TLRs = toll-like receptors; TUBB3 = Class III Beta-Tubulin.
