# Supplementary material for: Susceptibilities of CNS Cells towards Rabies Virus Infection Is Linked to Cellular Innate Immune Responses
Source: Viruses. 2022 Dec 29;15(1):88. doi: 10.3390/v15010088 (PMC9860954; doi:10.3390/v15010088)
Supplement: Supplementary file 1 [file viruses-15-00088-s001.zip › viruses-2013890-supplementary.pdf]

viruses MDPI

Supplementary material

Supplementary figures

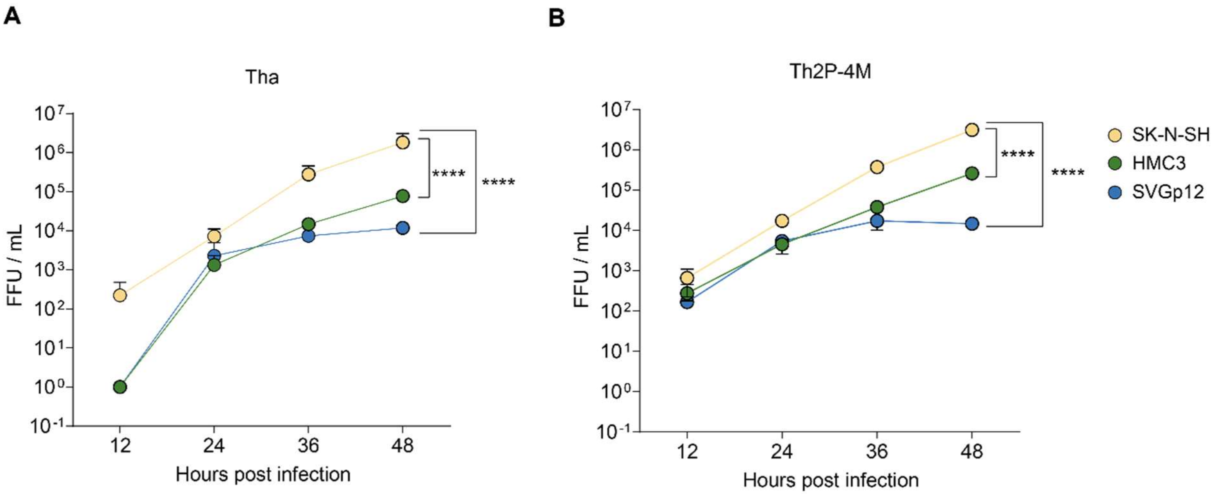

Figure S1. Comparative kinetics of Tha and Th2P-4M in SK-N-SH, HMC3, and SVGp12 cells. (A) Viral growth curve of Tha at 12-, 24-, 36-, and 48-hours post-infection. (B) Viral growth curve of Th2P-4M at 12-, 24-, 36-, and 48-hours post-infection. (A - B) Supernatants from SK-N-SH, SVGp12, and HMC3 infected with Tha or Th2P-4M at a MOI of 0.5 were titrated every 12 hours on BSR cells. Experiments were conducted three times (n=3) independently. Viral titers were compared with a Tukey's multiple comparison test (**** p-value<0.0001).

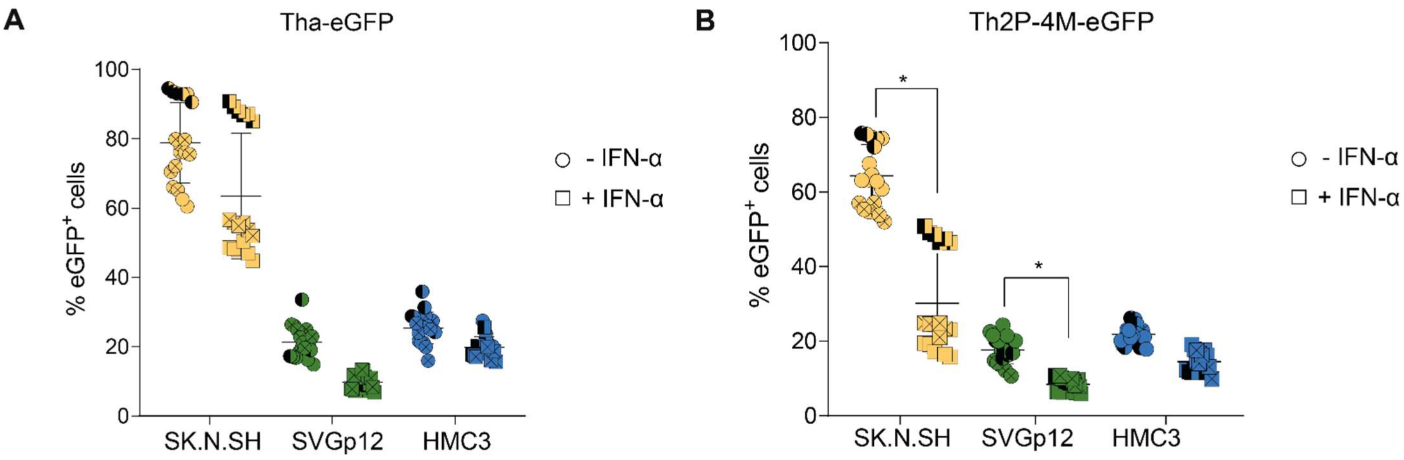

Figure S2. Influence of IFN-*α* treatment on eGFP expression in Tha-eGFP- and Th2P-eGFP-infected CNS cell lines. (A) Percentage of eGFP expressing cells in Tha-eGFP-infected SK.N.SH, SVGp12, and HMC3 at 48 hours post-infection. (B) Percentage of eGFP expressing cells in Th2P-4M-eGFP-infected SK.N.SH, SVGp12, and HMC3 at 48 hours post-infection. (A – B) Experiments were conducted three times (n=3) independently. Each dot represents imaging of one well of a 96-well-plate (approx. $8x10^3$ cells/well). Bars show mean ± SD. Different colours present different cell types and different symbol shapes indicate the three technical replicates performed. The percentages of $eGFP^+$ cells were analyzed using a mixed model with the replication factor as a random effect, followed by multiple comparisons corrected by Tukey's method (* adjusted p-value<0.0083). If no p-value is indicated, no significant difference was observed. eGFP = Enhanced Green Fluorescent Protein.

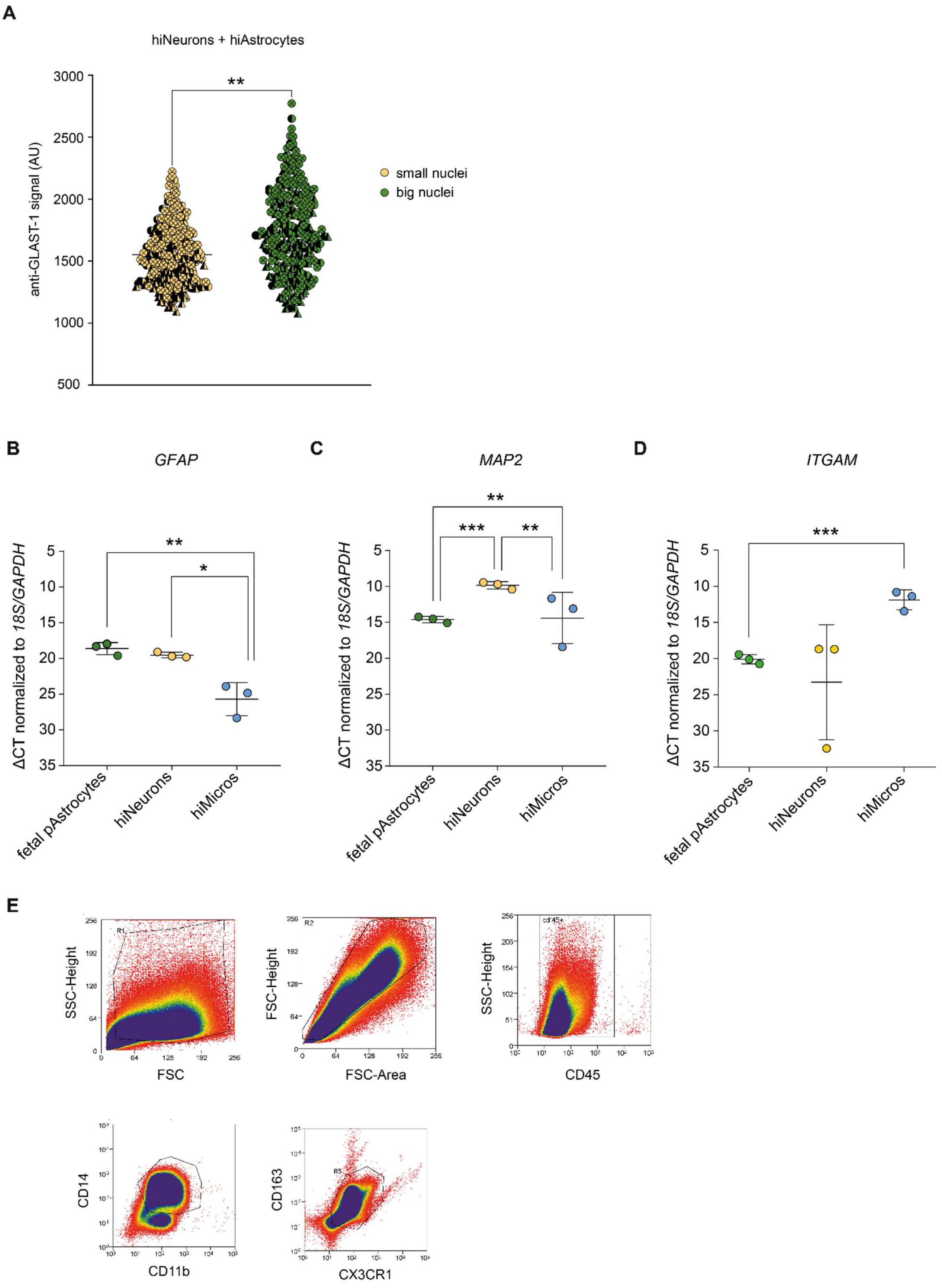

Figure S3. Validation of cellular fate by quantification of marker gene expression via RT-qPCR. (A) Cells in hiAstrocyte and hiNeurons co-cultures having a big nucleus (nuclei area>100 μm2) express significantly higher amounts of the

astrocyte-specific marker GLAST-1 compared to cells having a small nucleus (nuclei area≤100 μm2). Different colours present different cell types and different symbol shapes indicate the three technical replicates performed. Bars show mean ± SD which were compared with a two-tailed paired t-test (** p-value = 0.0057). More information on acquisition and analysis parameters can be found in S2 Table. (B) Fetal pAstrocytes and hiNeurons expressed higher levels of astrocyte-specific marker *GFAP* compared to hiMicros. (C) hiNeurons express higher levels of neuronal marker *MAP2* compared to fetal pAstrocytes and hiMicros. (D) hiMicros strongly express microglial marker *ITGAM* after co-culture with hiNeurons. Induced PSC were differentiated to hiMicros according to Takata et al [34]. (A – D) All experiments were performed three times (n=3) independently. (B - D) All bars show mean ± SD which were compared with an unpaired two-tailed t-test (**** p-value<0.0001, *** p-value<0.001, ** p-value<0.01, * p-value<0.05). (E) Fluorescent-activated cell sorting of hiMacs prior to co-culture with hiNeurons. After differentiation, 75% of hiMacs were expressing human CD45, CD11b, CD14, CD163, and CX3CR1. Human iMacs expressing human CD45, CD11b, CD14, CD163, and CX3CR1 were selected by using the MoFlo Astrios Flow cytometer (Beckman Coulter) and subsequently co-cultured with hiNeurons for differentiation into hiMicros. *GFAP* = Glial Fibrillary Acidic Protein; *ITGAM* = Integrin Subunit Alpha M; *MAP2* = Microtubule-associated Protein 2.

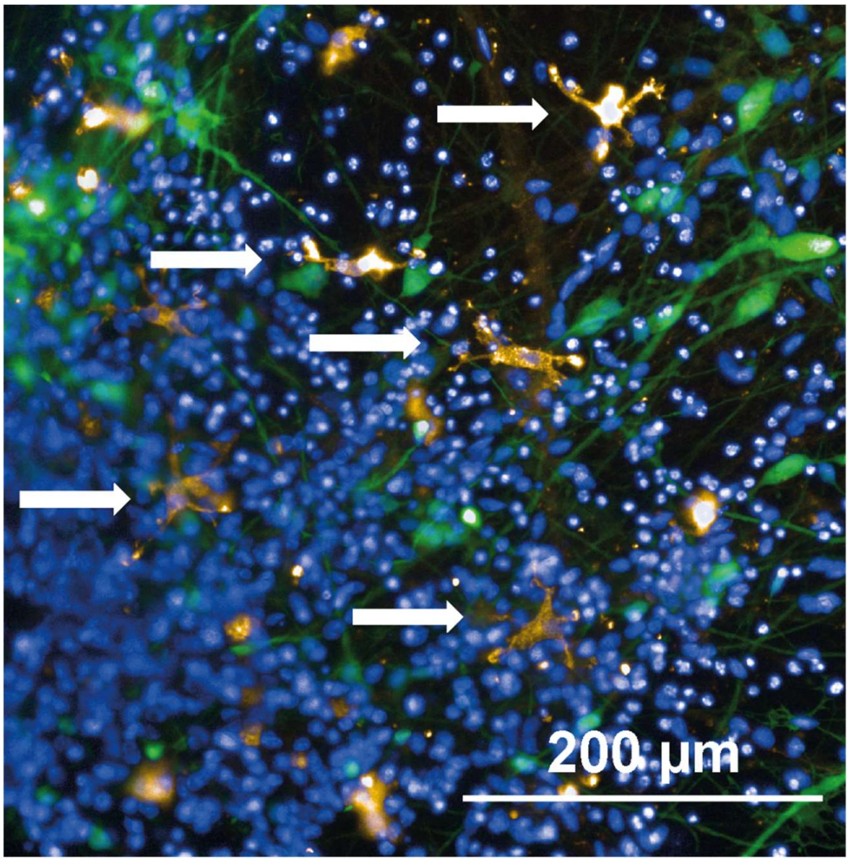

Figure S4. Detailed image of hiMicros (indicated with an arrow) in Th2P-4M-eGFP-infected cultures (MOI 0.5) consisting of hiNeurons, hhiAstrocytes, and hiMicroglia at 48 hours post-infection. Cells were infected with Tha-eGFP or Th2P-4M-eGFP (MOI 0.5) and imaged at 48 hours post-infection. All experiments were performed three times (n=3) independently. eGFP = Enhanced Green Fluorescent Protein; GLAST-1 = Glutamate Aspartate Transporter-1; IBA1 = Ionized calcium-binding adaptor molecule 1; TUBB3 = Class III Beta-Tubulin.

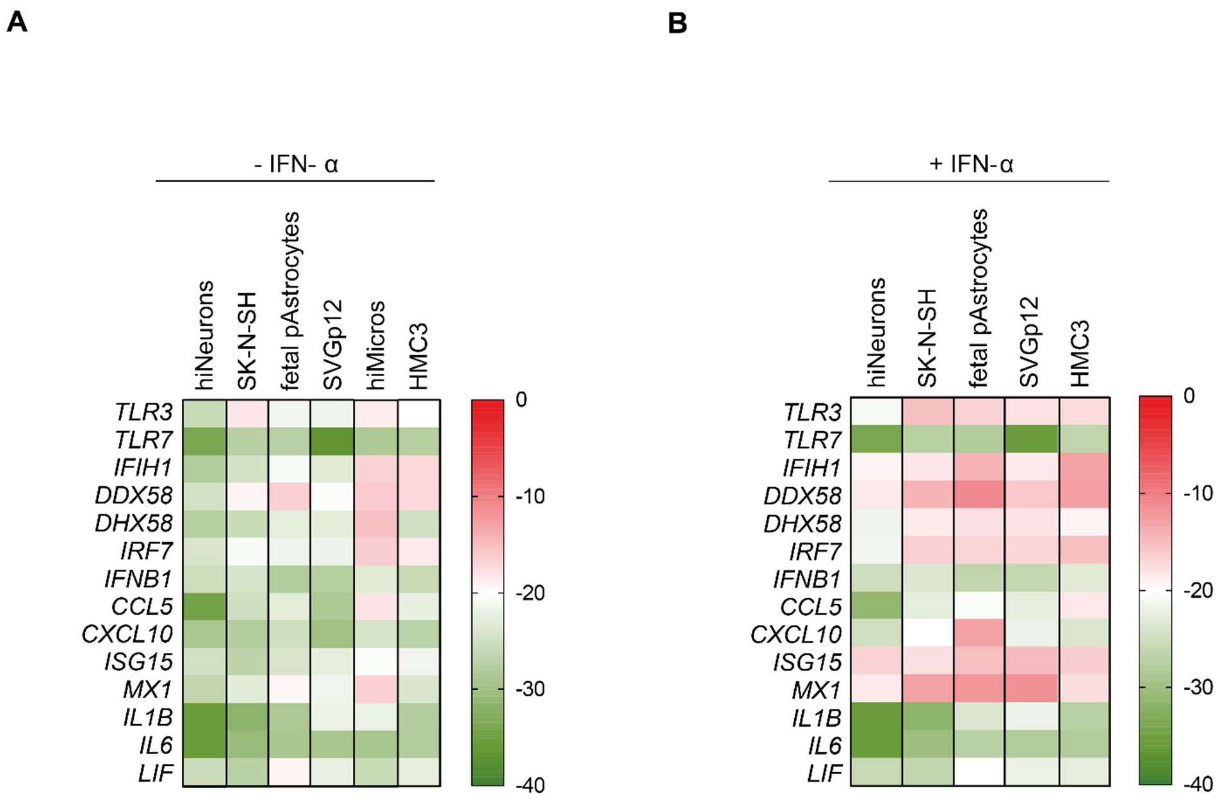

Figure S5. Basal expression of innate immunity genes in CNS cell types *in vitro*. (A) Basal gene expression of a panel of innate immunity genes of different CNS cell types. (B) Basal gene expression of a panel of innate immunity genes of different CNS cell types with subsequent IFN-α stimulation. (A-B) Innate immune gene expression (ΔCT) of non-infected cells quantified by qPCR which was normalized to the endogenous expression of housekeeping gene *18S*. All experiments were performed three times (n=3) independently. Heatmaps were generated using Prism (version 9). The scale presents negative transformed ΔCT (ΔCT x -1) values.

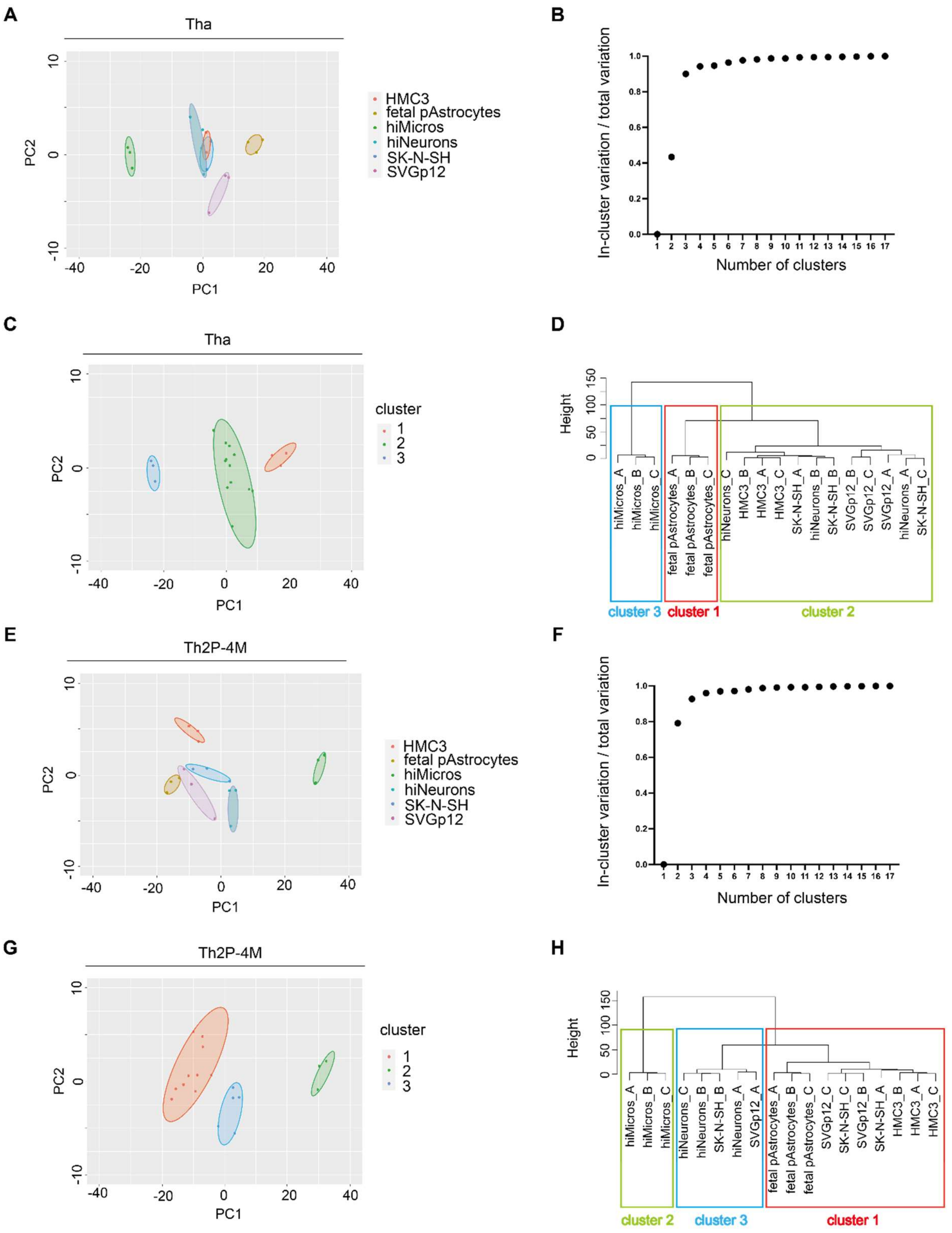

S6 Fig. Innate immune responses of CNS cell types upon Tha and Th2P-4M infection (without IFN-α treatment). (A) Principal component analysis of the distinct cellular innate immune responses towards Tha infection. (B) Calculation of the relative in-cluster variation in cellular innate immune responses upon Tha infection revealed three distinct clusters. The number of clusters was determined based on calculating the within cluster variability. (C) The same principal

components as in A but coloured according to the clusters found with hierarchical clustering. (D) hierarchical clustering based on the Euclidean distance with a **** linkage of three distinct cellular innate immune responses upon Tha infection. (E) Principal component analysis of the distinct cellular innate immune responses towards Th2P-4M infection. (F) Calculation of the relative in-cluster variation in cellular innate immune responses upon Th2P-4M infection revealed three distinct clusters. The number of clusters was determined based on calculating the minimal in-cluster variation. (G) Principal component analysis of three distinct cellular innate immune responses towards Th2P-4M infection. (H) Euclidian clustering of three distinct cellular innate immune responses upon Th2P-4M infection. (A-H) Cells were infected with Tha or Th2P-4M (MOI 5) and gene expression was quantified at 48 hours post-infection via qPCR. All experiments were performed three times (n=3) independently. For all calculations or schematic presentations, gene expression (ΔΔCT) was normalized to the expression of the housekeeping gene *18S* and the respective non-infected mock. PCA, Euclidian clustering and calculation of the relative in-cluster variation was performed using R (version 4.0.4).

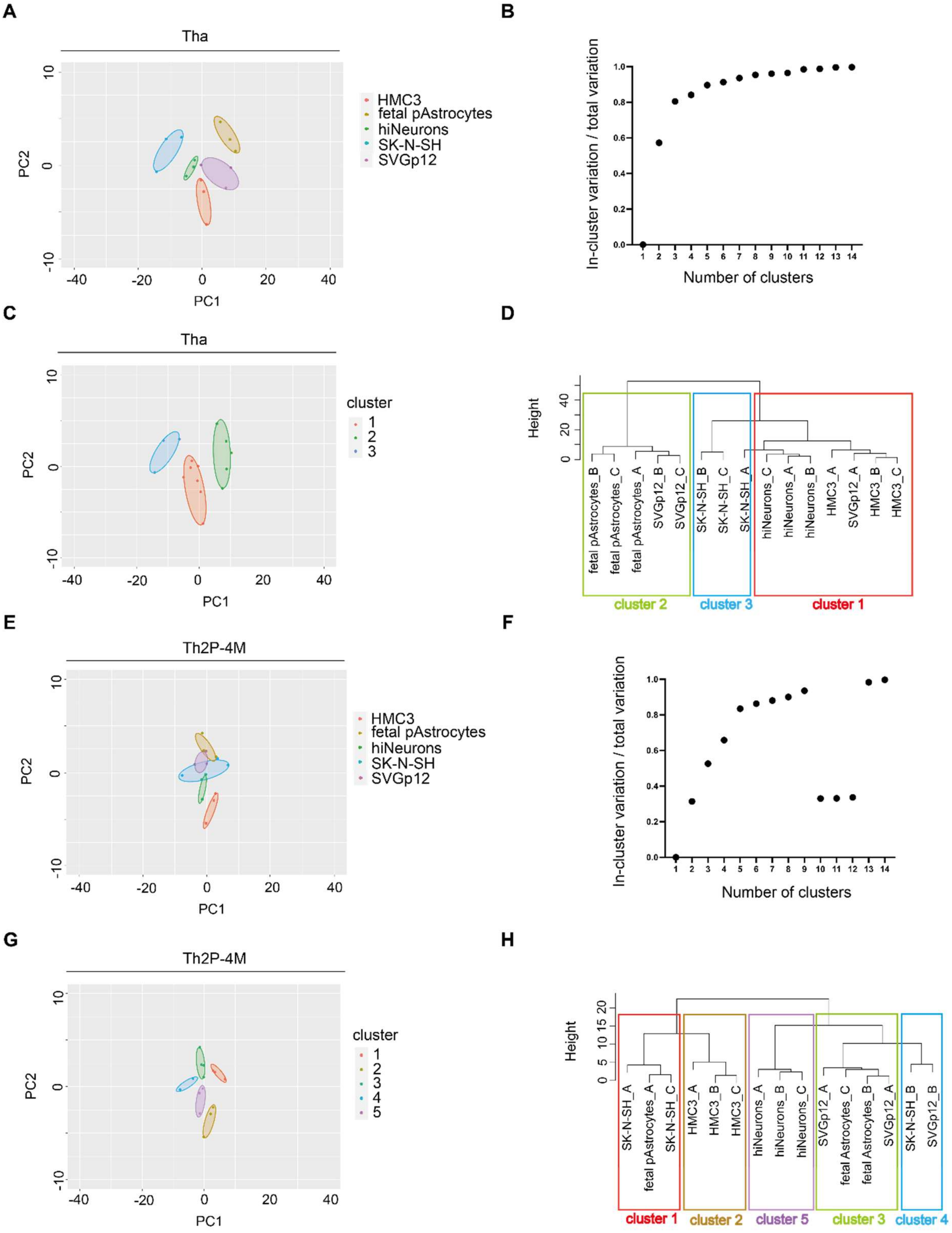

Figure S7. Innate immune responses of CNS cell types upon Tha and Th2P-4M infection with subsequent IFN-α treatment. (A) Principal component analysis of the distinct cellular innate immune responses towards Tha infection (+ IFN-α treatment). (B) Calculation of the relative in-cluster variation in cellular innate immune responses upon Tha infection revealed three distinct clusters (+ IFN-α treatment). The number of clusters was determined based on

calculating the within cluster variability. (C) The same principal components as in A but coloured according to the clusters found with hierarchical clustering. (D) hierarchical clustering based on the Euclidean distance with a **** linkage. (E) Principal component analysis of the distinct cellular innate immune responses towards Th2P-4M infection with subsequent IFN-α treatment. (F) Calculation of the relative in-cluster variation in cellular innate immune responses upon Th2P-4M infection revealed five distinct clusters with subsequent IFN-α treatment. The number of clusters was determined based on calculating the minimal in-cluster variation. (G) Principal component analysis of five distinct cellular innate immune responses towards Th2P-4M infection with subsequent IFN-α treatment. (H) Euclidian clustering of three distinct cellular innate immune responses upon Th2P-4M infection with subsequent IFN-α treatment. (A-H) Cells were infected with Tha or Th2P-4M (MOI 5) and treated at 24 hours post-infection with IFN-α. Gene expression was quantified at 48 hours post-infection via qPCR. All experiments were performed three times (n=3) independently. For all calculations or schematic presentations, gene expression (ΔΔCT) was normalized to the expression of the housekeeping gene *18S* and the respective non-infected mock. PCA, Euclidian clustering and calculation of the relative in-cluster variation was performed using R (version 4.0.4).

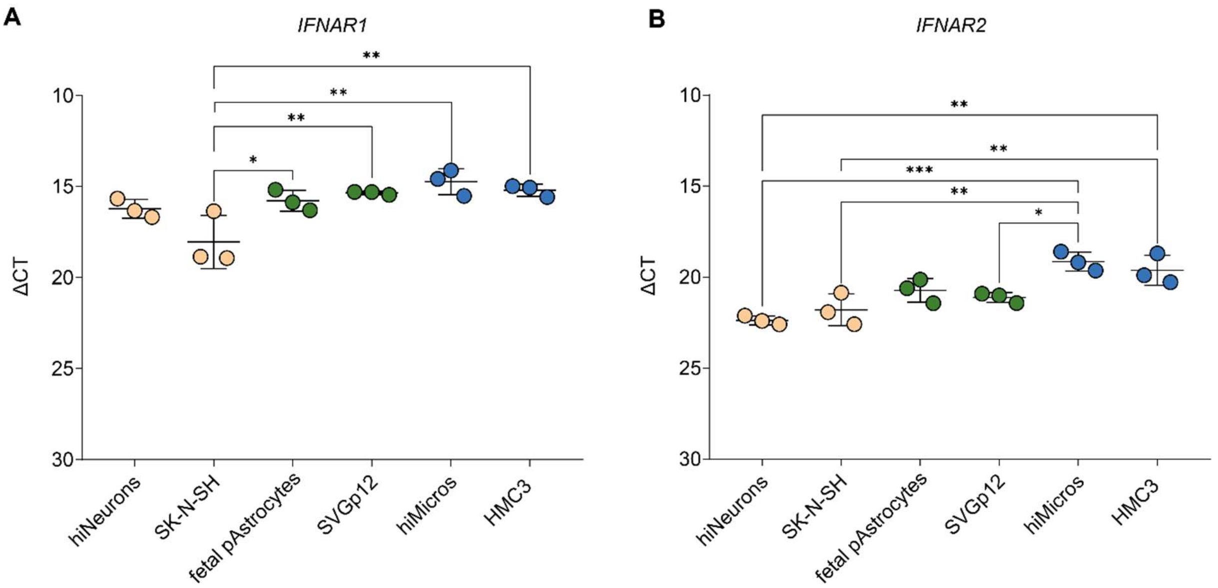

Figure S8. Expression of human interferon receptors (*IFNAR1* and *IFNAR2*) in neurons, astrocytes, and microglia. (A) Human *IFNAR1* expression in the different CNS cell types. (B) Human *IFNAR2* expression in the different CNS cell types. (A – B) Gene expression of human *IFNAR1* and *IFNAR2* was quantified at 48 hours after seeding. All bars show mean ± SD with a Tukey's multiple comparisons test (*** adjusted p-value<0.001, ** adjusted p-value<0.01, * adjusted p-value<0.05).

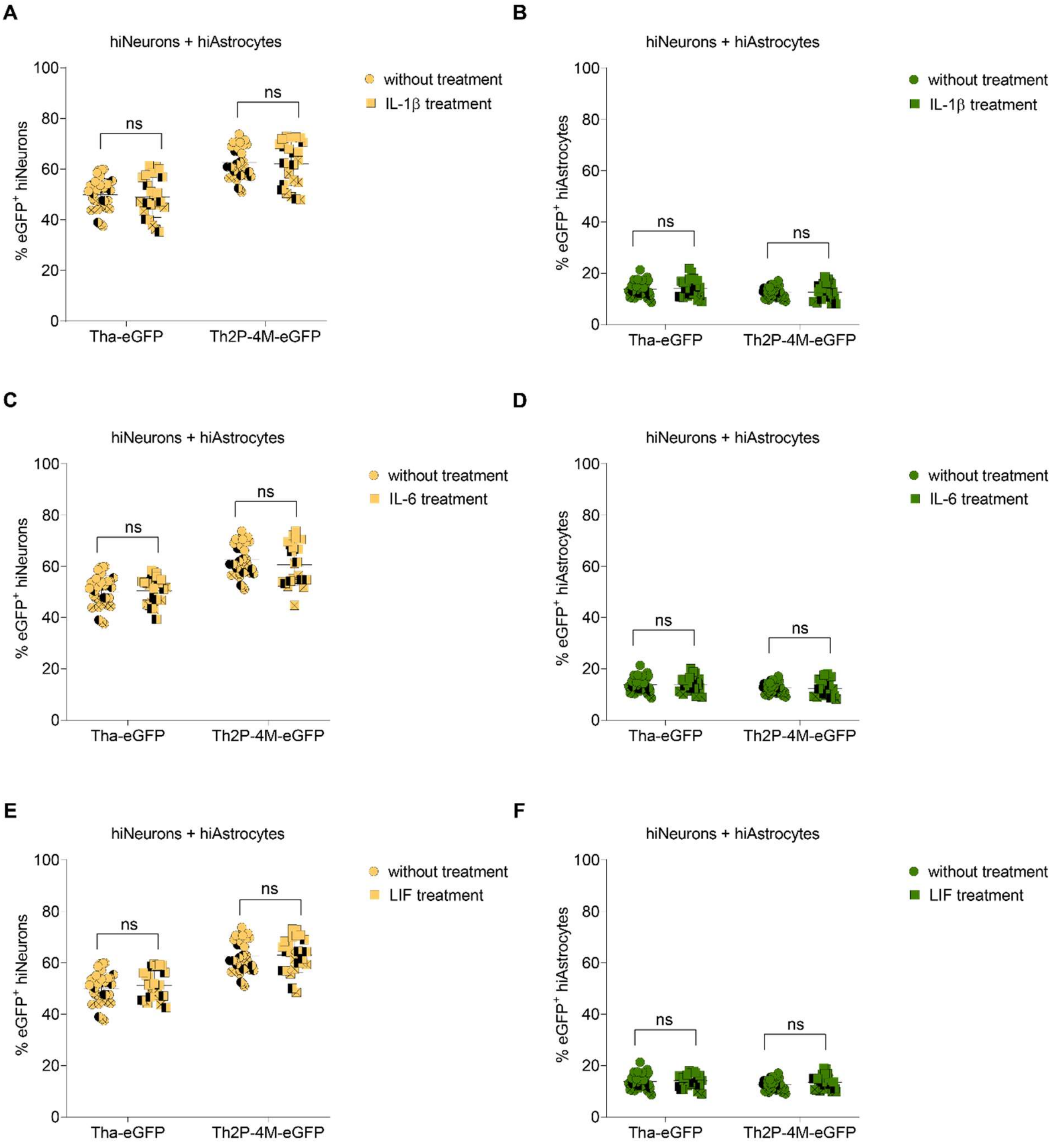

Figure S9. Neither IL-1β, IL-6, nor LIF protect human hiNeurons or hiAstrocytes from Tha-eGFP or Th2P-4M-eGFP infection *in vitro*. (A) Quantification of eGFP+ hiNeurons in co-cultures consisting of hiNeurons and hiAstrocytes upon treatment with recombinant human IL-1β. (B) Quantification of eGFP+ hiAstrocytes in co-cultures consisting of hiNeurons and hiAstrocytes upon treatment with recombinant human IL-1β. (C) Quantification of eGFP+ hiNeurons in co-cultures consisting of hiNeurons and hiAstrocytes upon treatment with recombinant human IL-6. (D) Quantification of eGFP+ hiAstrocytes in co-cultures consisting of hiNeurons and hiAstrocytes upon treatment with recombinant human IL-6. (E) Quantification of eGFP+ hiNeurons in co-cultures consisting of hiNeurons and hiAstrocytes upon treatment with recombinant human LIF. (F) Quantification of eGFP+ hiAstrocytes in co-cultures consisting of hiNeurons and hiAstrocytes upon treatment with recombinant human LIF. (A-F) Prior to infection, cultures consisted of 86% hiNeurons and 14% hiAstrocytes (S6 Table). Cells were infected with Tha-eGFP or Th2P-4M-eGFP (MOI 0.5) and imaged at 48

hours post-infection. Two hours post-infection, the culture medium was removed, and cells were treated with 100 ng/mL recombinant human 1β, IL-6 or LIF. All experiments were performed three times (n=3) independently. Each dot represents imaging of one well of a 96-well-plate (approx. $1x10^4$ cells/well). Bars show mean ± SD. Different colours present different cell types and different symbol shapes indicate the different treatment conditions and the three technical replicates performed. The percentages of $eGFP^+$ cells or $eGFP^+$ $TUBB3^+$ cells were analyzed using a mixed model with the replication factor as a random effect, followed by multiple comparisons corrected by Tukey's method. IL-1β = interleukin 1 beta; IL-6 = interleukin 6; LIF = leukemia inhibitory factor; ns = non-significant.

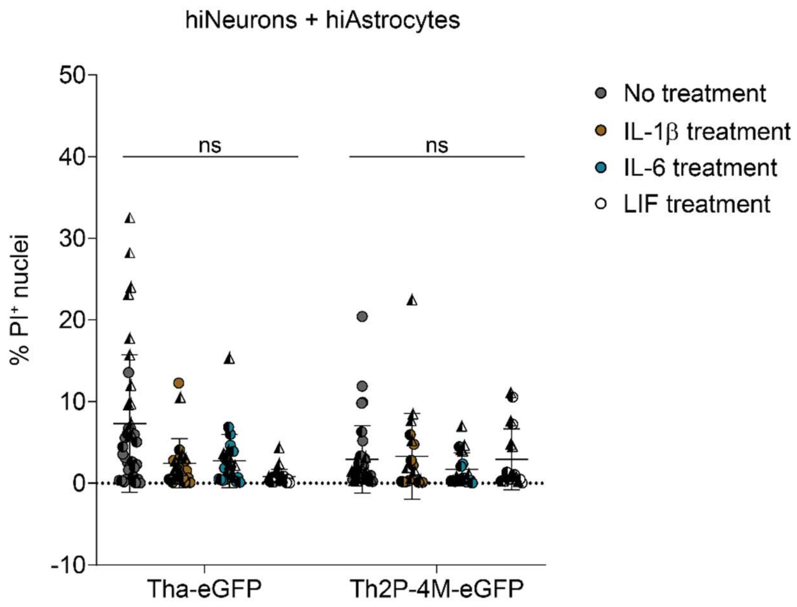

Figure S10. Neither IL-1β, IL-6 nor LIF significantly modulates the percentage of PI$^+$ hNSC-derived hiNeurons and hiAstrocytes upon Tha-eGFP or Th2P-4M-eGFP infection. Cell death was measured by quantifying the percentage of PI$^+$ cells by fluorescence microscopy. Different colours present different treatment conditions and different symbol shapes indicate the three technical replicates performed. All bars show mean ± SD with a Tukey's multiple comparisons test (**** adjusted p-value<0.0001, *** adjusted p-value<0.001, ** adjusted p-value<0.01, * adjusted p-value<0.05). PI$^+$ = positive staining for propidium iodine

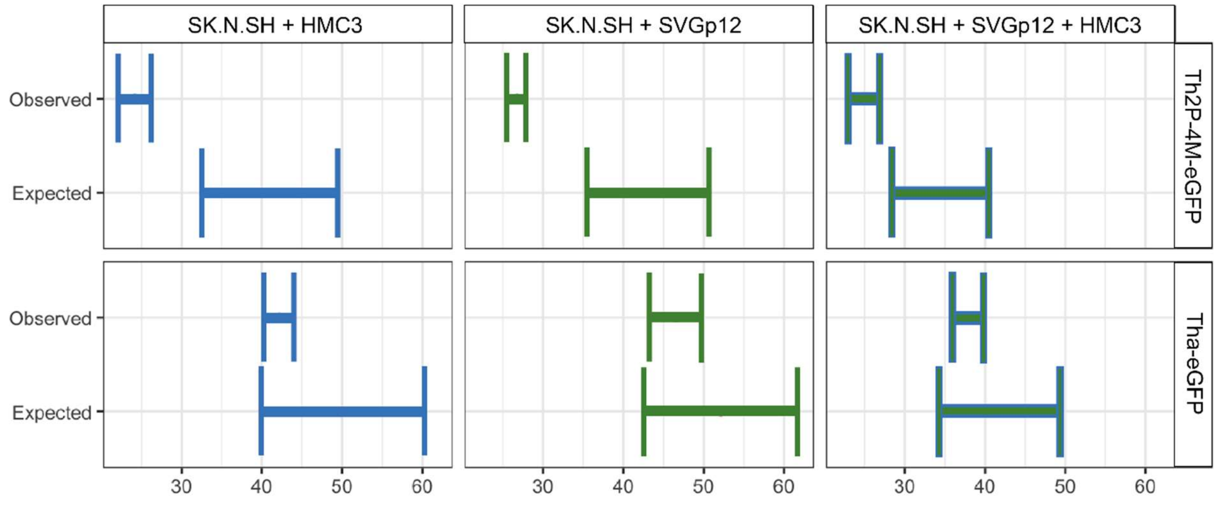

Figure S11. Statistical comparison of confidence intervals between observed and expected quantification of eGFP⁺ cells in co-cultures. The expected CI_{95} of eGFP⁺ cells in co-cultures were calculated bases on a Student distribution under the hypothesis that there is no interaction between the infection and the environment. Since the cells in co-cultures (Fig 5A) were mixed in equal ratios, the environment is composed of 50% of cells type and 50% of cells type B, allowing us the calculate the expected percentage of eGFP⁺ cells according to the following formula: the proportion of infected cells is expected to be the mean of (1) the percentage of infected cells in the monoculture with cell type A and (2) the percentage of infected cells in the monoculture with cell type B. A detailed description of the analysis can be found in the Materials and Methods section. The expected (based on the previously mentioned calculation based on values illustrated in Fig 1C) CI_{95} of eGFP⁺ cells in Th2P-4M-eGFP-infected co-cultures did not overlap with the observed CI_{95} of Th2P-4M-eGFP-infected co-cultures (Fig 5A). In Tha-eGFP-infected co-cultures, however, expected (calculated based on values illustrated in Fig 1C) CI_{95} of eGFP⁺ cells overlapped with the observed CI_{95} of total Tha-eGFP-infected cells in co-cultures (Fig 5A). Calculations were based on data acquired from monoculture experiments (Fig 1C) which were conducted three times (n=3) independently assuming that cells were mixed in equal ratios. Calculations and graphical representations were performed using R (version 4.0.4). CI_{95} = 95% confidence interval.

Supplementary tables

Table S1. Antibodies used for fluorescence microscopy. GLAST-1 = Glutamate Aspartate Transporter-1; Acidic Protein; IBA1 = Ionized calcium binding adaptor molecule 1; TUBB3 = Class III Beta-Tubulin.

Antibody	Marker for	Origin	Dilution	Reference
**Anti-TUBB3**	Neurons	Mouse	1:1000	Sigma T858
**Anti-GLAST**	Astrocytes	Rabbit	1:500	Invitrogen PA5-111080
**Anti-IBA1**	Microglia	Rabbit	1:100	Proteintech 10904-1-AP
**DyLight 549**	Anti-mouse	Goat	1 :1000	KPL 072-04-18-06
**AlexaFluor 594**	Anti-rabbit	Donkey	1 :1000	Invitrogen A21207
**Hoechst 33342**	Nucleus	-	1 :10 000	Thermo Fisher H1399
**Propidium iodine**	Dead nuclei	-	-	Thermo Fisher, R37610
**Nuc® Blue Live**	Living nuclei	-	-	Thermo Fisher, R37610

Table S2. Technical parameter used for image acquisition by OPERA Phenix High Content Screening (Perkin Elmer) and subsequent analysis by Columbus Analysis Software (Perkin Elmer). eGFP = Enhanced green fluorescent protein; IBA1 = Ionized calcium binding adaptor molecule 1; PI = Propidium iodide, TUBB3 = beta-(III)-tubulin; TUNEL = Terminal deoxynucleotidyl transferase dUTP nick end labeling; TRITC = Tetramethylrhodamine. *All values present relative intensity levels (arbitrary units) except for values marked with an asterisk which refer to the surface area.

Measurement	Staining	Channel	Acquisition parameters	Selection criteria thresholds (Columbus Analysis Software)
**PI⁺ SK-N-SH**	PI	TRITC (561/570-630)	300 msec 100 %	PI > 600
**PI⁺ SVGp12**	PI	TRITC (561/570-630)	300 msec 100 %	PI>1500
**PI⁺ HMC3**	PI	TRITC (561/570-630)	300 msec 100 %	PI>1250
**PI⁺ SK-N-SH + SVGp12 + HMC3**	PI	TRITC (561/570-630)	300 msec 100 %	PI>1250
**TUNEL⁺ cells**	TUNEL	Cy3 (561/570-630)	300 msec 100 %	TUNEL>1000
**eGFP⁺ cells**	eGFP	eGFP (488/500-550)	200 msec 50 %	eGFP>500
**TUBB3⁺ cells**	TUBB3	Alexa 594 (561/570-630)	600 msec 100 %	TUBB3⁺>350
**IBA1⁺ cells**	IBA1	Alexa 594 (561/570-630)	600 msec 100 %	IBA1⁺>500
**GLAST⁺ cells**	GLAST	Alexa 594 (561/570-630)	600 msec 100 %	GLAST⁺>500
**Small nuclei**	Hoechst	Hoechst (375/435 - 480)	50 msec 100 %	* Hoechst≤100 μm^2
**Big nuclei**	Hoechst	Hoechst (375/435 - 480)	50 msec 100 %	* Hoechst>100 μm^2

Table S3. Primer sequences used to measure human gene expression via RT-qPCR primers. fwd = forward primer; rev = reverse primer.

Gene	Sense	Gene name and primer sequence	Reference/Source
18S	fwd	18 Svedberg ribosomal RNA<br>5′-TGCGAATGGCTCATTAAATCAGT-3′	Eurofins
	rev	18 Svedberg ribosomal RNA<br>5′-AGAGGAGCGAGCGACCAA-3′	
CCL5	fwd and rev	C-C Motif Chemokine Ligand 5	QT00090083, Qiagen
CXCL10	fwd and rev	C-X-C Motif Chemokine Ligand 10	QT01003065, Qiagen
DDX58	fwd and rev	DExD/H-Box Helicase 58	QT00040509, Qiagen
IFIH1	fwd and rev	Interferon Induced With Helicase C Domain 1	QT00033789, Qiagen
ITGAM	fwd	Integrin Subunit Alpha M<br>(5′-GAAAGGCAAGGAAGCCGGAG -3′)	[75],<br>Eurofins
	rev	Integrin Subunit Alpha M<br>(5′-TGGATCTGTCCTTCTCTTAGCCG-3′)	
IFNAR1	fwd and rev	Interferon receptor 1	QT00094017
IFNAR2	fwd and rev	Interferon receptor 2	QT00102844
GAPDH	fwd	Glyceraldehyde-3-Phosphate Dehydrogenase<br>5′-GAAGGTGAAGGTCGGAGT-3′	Eurofins
	rev	Glyceraldehyde-3-Phosphate Dehydrogenase<br>5′-GGTCATGAGTCTTCCACGAT-3′	Eurofins
GFAP	fwd and rev	Glial Fibrillary Acidic Protein	QT02403093, Qiagen
IFNB1	fwd	Interferon Beta 1<br>5′ – TCTTTCCATGAGCTACAACTT-3′	Eurofins
	rev	Interferon Beta 1<br>5′ –GCAGTATTCAAGCCTCCCATTC-3′	
IL1B	fwd and rev	Interleukin 1 beta	QT00021385, Qiagen
IL6	fwd and rev	Interleukin 6	QT00083720, Qiagen
IRF7	fwd and rev	Interferon Regulatory Factor 7	QT00210595, Qiagen
ISG15	fwd and rev	Interferon-stimulated gene 15	QT00072814, Qiagen
LIF	fwd and rev	Leukemia inhibitory factor	QT00001442, Qiagen
MAP2	fwd and rev	Microtubule Associated Protein 2	QT00057358, Qiagen
MX1	fwd	Interferon-induced GTP-binding protein Mx1<br>5′-CCTTTCTTCAGGTGGAACAC- 3′	Eurofins [26]
	rev	Interferon-induced GTP-binding protein Mx1	

		5′-GACAGGACCATCGGAATCTTG-3′	
DHX58	fwd and rev	Laboratory of genetics and physiology 2	QT01051148, Qiagen
TLR3	fwd and rev	Toll-like receptor 3	QT00007714, Qiagen
TLR7	fwd and rev	Toll-like receptor 7	QT00030030, Qiagen

Table S4. Determination of percentage of hiNeurons (=small nuclei) and hiAstrocytes (=big nuclei) after neural differentiation of human neural stem cells for 21 days. Cells having a small nucleus comprised cells with a nuclear area≤100 μm^2. Cells presenting a big nucleus comprised cells with a nuclear area>100 μm^2. Twenty-one days after the induction of neural differentiation, cultures comprised on average 85.6% hiNeurons (small nuclei) and 14.4% hiAstrocytes (big nuclei). Data were collected from three (n=3) independently performed experiments acquiring information from approx. 1.43×10^6 cells. Every row in the table presents acquisition of one complete well of a 96-well-plate. Acquisition and analysis parameters are described in S2 Table. IBA1 = ionized calcium-binding adapter molecule 1; No = Number.

No. of small nuclei	No. of big nuclei	No. of total nuclei	Proportion of hiNeurons (% small nuclei)	Proportion of hiAstrocytes (% big nuclei)
29906	3691	33597	89.01	10.99
32435	5420	37855	85.68	14.32
30801	4771	35572	86.59	13.41
28895	7493	36388	79.41	20.59
31333	3198	34531	90.74	9.26
32717	4457	37174	88.01	11.99
29983	5541	35524	84.40	15.60
24985	7718	32703	76.40	23.60
25859	3273	29132	88.76	11.24
32736	5196	37932	86.30	13.70
33585	5133	38718	86.74	13.26
29814	7038	36852	80.90	19.10
18476	3586	22062	83.75	16.25
23837	4204	28041	85.01	14.99
23516	5156	28672	82.02	17.98
27855	7236	35091	79.38	20.62
19611	3106	22717	86.33	13.67
23689	5400	29089	81.44	18.56
24917	6123	31040	80.27	19.73
21451	6662	28113	76.30	23.70

19913	2050	21963	90.67	9.33
20280	3360	23640	85.79	14.21
24816	2909	27725	89.51	10.49
15480	4138	19618	78.91	21.09
17317	2603	19920	86.93	13.07
20145	3326	23471	85.83	14.17
10207	2076	12283	83.10	16.90
18000	3442	21442	83.95	16.05
14831	3479	18310	81.00	19.00
14241	2777	17018	83.68	16.32
10982	1535	12517	87.74	12.26
13068	2925	15993	81.71	18.29
18115	2587	20702	87.50	12.50
17647	2427	20074	87.91	12.09
9547	1525	11072	86.23	13.77
15468	2222	17690	87.44	12.56
8848	1726	10574	83.68	16.32
9617	1671	11288	85.20	14.80
6241	1138	7379	84.58	15.42
10525	1863	12388	84.96	15.04
15240	2476	17716	86.02	13.98
15790	1946	17736	89.03	10.97
10585	1055	11640	90.94	9.06
16935	2605	19540	86.67	13.33
17496	2749	20245	86.42	13.58
14595	2415	17010	85.80	14.20
11587	1404	12991	89.19	10.81
14358	2283	16641	86.28	13.72
19000	3102	22102	85.97	14.03
19100	2668	21768	87.74	12.26
14245	2123	16368	87.03	12.97
13261	2802	16063	82.56	17.44
13532	2095	15627	86.59	13.41
15096	2156	17252	87.50	12.50
11508	1530	13038	88.27	11.73
12433	2473	14906	83.41	16.59
14757	3357	18114	81.47	18.53
14946	3224	18170	82.26	17.74
10840	1368	12208	88.79	11.21
9681	2493	12174	79.52	20.48
12767	2950	15717	81.23	18.77
14627	3331	17958	81.45	18.55

12988	1751	14739	88.12	11.88
10323	2770	13093	78.84	21.16
11254	1491	12745	88.30	11.70
10191	1275	11466	88.88	11.12
9361	942	10303	90.86	9.14
10121	1346	11467	88.26	11.74

Table S5. Determination of percentage of hiNeurons (=small nuclei), hiAstrocytes (=big nuclei), and hiMicros (IBA1⁺) in human stem cell-derived CNS cell triple cultures. hiNeurons were defined as cells with a nuclear area≤100 μm^2 (small nuclei). hiAstrocytes were defined as cells with a nuclear area>100 μm^2 (big nuclei) and negative staining for IBA1. Validation of these parameters can be found in S4A Fig. hiMicros were identified by positive staining for IBA1. Three weeks after co-culture, cultures comprised on average 71.0% hiNeurons (small nuclei), 18.4% hiAstrocytes (big nuclei), and 10.6% hiMicros (IBA1$^+$). Data were collected from three (n=3) independently performed experiments acquiring information from approx. 2.96x10^6 cells. Every row in the table presents acquisition of one complete well of a 96-well-plate. Acquisition and analysis parameters are described in S2 Table. IBA1 = ionized calcium-binding adapter molecule 1; No = number.

No. of hiMicros	No. of small nuclei	No. of big nuclei	No. of big nuclei – microglia	No. of total nuclei	Proportion of hiNeurons (% small nuclei)	Proportion of hiAstrocytes (% big nuclei and IBA$^-$)	Proportion of hiMicros (% IBA$^+$)
868	22337	7183	6315	29520	75.67	21.39	2.94
934	23345	8266	7332	31611	73.85	23.19	2.95
1119	24799	8114	6995	32913	75.35	21.25	3.40
1275	23676	7255	5980	30931	76.54	19.33	4.12
877	24214	8255	7378	32469	74.58	22.72	2.70
960	24180	7270	6310	31450	76.88	20.06	3.05
730	20213	4466	3736	24679	81.90	15.14	2.96
773	23602	6381	5608	29983	78.72	18.70	2.58
1043	23706	9013	7970	32719	72.45	24.36	3.19
1009	21256	6625	5616	27881	76.24	20.14	3.62
840	24548	7384	6544	31932	76.88	20.49	2.63
907	30822	8473	7566	39295	78.44	19.25	2.31
1017	21053	8584	7567	29637	71.04	25.53	3.43
931	22413	8448	7517	30861	72.63	24.36	3.02
745	24401	7889	7144	32290	75.57	22.12	2.31
730	26366	8499	7769	34865	75.62	22.28	2.09
1105	20101	7685	6580	27786	72.34	23.68	3.98
1400	19406	5825	4425	25231	76.91	17.54	5.55
708	27480	7231	6523	34711	79.17	18.79	2.04
1480	23647	8653	7173	32300	73.21	22.21	4.58
932	16484	5049	4117	21533	76.55	19.12	4.33
713	21354	6530	5817	27884	76.58	20.86	2.56

874	22755	6902	6028	29657	76.73	20.33	2.95
844	24749	7803	6959	32552	76.03	21.38	2.59
715	57377	7748	7033	65125	88.10	10.80	1.10
990	17687	6124	5134	23811	74.28	21.56	4.16
835	14254	4360	3525	18614	76.58	18.94	4.49
958	24637	8735	7777	33372	73.83	23.30	2.87
1684	19616	7528	5844	27144	72.27	21.53	6.20
854	24760	8270	7416	33030	74.96	22.45	2.59
1159	22328	8669	7510	30997	72.03	24.23	3.74
1026	25953	8429	7403	34382	75.48	21.53	2.98
904	20807	6543	5639	27350	76.08	20.62	3.31
494	23493	7154	6660	30647	76.66	21.73	1.61
610	25195	7059	6449	32254	78.11	19.99	1.89
632	24594	7573	6941	32167	76.46	21.58	1.96
724	30258	7863	7139	38121	79.37	18.73	1.90
728	26801	8011	7283	34812	76.99	20.92	2.09
625	21392	5197	4572	26589	80.45	17.20	2.35
730	29093	7049	6319	36142	80.50	17.48	2.02
664	29061	7273	6609	36334	79.98	18.19	1.83
792	20383	6908	6116	27291	74.69	22.41	2.90
465	25877	5296	4831	31173	83.01	15.50	1.49
605	25105	6234	5629	31339	80.11	17.96	1.93
749	23110	7043	6294	30153	76.64	20.87	2.48
1242	14731	6253	5011	20984	70.20	23.88	5.92
697	23463	6069	5372	29532	79.45	18.19	2.36
670	23008	6140	5470	29148	78.94	18.77	2.30
795	6905	1411	616	8316	83.03	7.41	9.56
1279	13745	6009	4730	19754	69.58	23.94	6.47
539	19037	5046	4507	24083	79.05	18.71	2.24
489	22859	5618	5129	28477	80.27	18.01	1.72
528	9032	2741	2213	11773	76.72	18.80	4.48
870	12350	3299	2429	15649	78.92	15.52	5.56
604	7836	2913	2309	10749	72.90	21.48	5.62
522	9730	3127	2605	12857	75.68	20.26	4.06
1197	16670	7725	6528	24395	68.33	26.76	4.91
762	22621	8016	7254	30637	73.84	23.68	2.49
573	22210	5627	5054	27837	79.79	18.16	2.06
792	23169	7126	6334	30295	76.48	20.91	2.61
3640	22135	6215	2575	28350	78.08	9.08	12.84
807	21415	6455	5648	27870	76.84	20.27	2.90
455	18805	5013	4558	23818	78.95	19.14	1.91
491	21417	6718	6227	28135	76.12	22.13	1.75

853	1847	1069	216	2916	63.34	7.41	29.25
1217	2470	2552	1335	5022	49.18	26.58	24.23
786	5003	1602	816	6605	75.75	12.35	11.90
841	4984	2032	1191	7016	71.04	16.98	11.99
674	5135	2241	1567	7376	69.62	21.24	9.14
474	8864	2869	2395	11733	75.55	20.41	4.04
693	7589	2980	2287	10569	71.80	21.64	6.56
1059	2559	1940	881	4499	56.88	19.58	23.54
735	6276	2125	1390	8401	74.71	16.55	8.75
872	5802	2294	1422	8096	71.67	17.56	10.77
827	6708	2430	1603	9138	73.41	17.54	9.05
877	5420	2875	1998	8295	65.34	24.09	10.57
516	6625	2965	2449	9590	69.08	25.54	5.38
434	10028	3404	2970	13432	74.66	22.11	3.23
444	11131	3667	3223	14798	75.22	21.78	3.00
740	9312	2672	1932	11984	77.70	16.12	6.17
931	8894	2603	1672	11497	77.36	14.54	8.10
955	8631	2893	1938	11524	74.90	16.82	8.29
818	6624	2286	1468	8910	74.34	16.48	9.18
471	11527	2829	2358	14356	80.29	16.43	3.28
638	9975	2957	2319	12932	77.13	17.93	4.93
357	12840	3129	2772	15969	80.41	17.36	2.24
1469	2163	2067	598	4230	51.13	14.14	34.73
789	7337	2648	1859	9985	73.48	18.62	7.90
771	8583	2926	2155	11509	74.58	18.72	6.70
668	6495	2181	1513	8676	74.86	17.44	7.70
822	6197	2733	1911	8930	69.40	21.40	9.20
480	10526	3856	3376	14382	73.19	23.47	3.34
789	11678	3658	2869	15336	76.15	18.71	5.14
545	10513	3893	3348	14406	72.98	23.24	3.78
1023	2017	1346	323	3363	59.98	9.60	30.42
761	7519	3161	2400	10680	70.40	22.47	7.13
769	4597	2496	1727	7093	64.81	24.35	10.84
865	5300	2940	2075	8240	64.32	25.18	10.50
600	4454	2770	2170	7224	61.66	30.04	8.31
576	5884	3539	2963	9423	62.44	31.44	6.11
536	6043	3107	2571	9150	66.04	28.10	5.86
382	6898	3373	2991	10271	67.16	29.12	3.72
1108	2613	1171	63	3784	69.05	1.66	29.28
706	4256	2524	1818	6780	62.77	26.81	10.41
558	6233	3112	2554	9345	66.70	27.33	5.97
580	7317	3121	2541	10438	70.10	24.34	5.56

522	5028	3364	2842	8392	59.91	33.87	6.22
593	4614	3250	2657	7864	58.67	33.79	7.54
328	7979	4009	3681	11988	66.56	30.71	2.74
178	8175	3629	3451	11804	69.26	29.24	1.51
886	7886	2957	2071	10843	72.73	19.10	8.17
1108	9542	2940	1832	12482	76.45	14.68	8.88
1164	7174	2832	1668	10006	71.70	16.67	11.63
650	4868	2317	1667	7185	67.75	23.20	9.05
877	6150	2692	1815	8842	69.55	20.53	9.92
630	10736	3239	2609	13975	76.82	18.67	4.51
367	15722	3689	3322	19411	81.00	17.11	1.89
1311	2845	1610	299	4455	63.86	6.71	29.43
1283	2730	1650	367	4380	62.33	8.38	29.29
1372	2904	1511	139	4415	65.78	3.15	31.08
1593	4602	5662	4069	10264	44.84	39.64	15.52
1195	2795	2113	918	4908	56.95	18.70	24.35
1044	5635	3406	2362	9041	62.33	26.13	11.55
972	4236	2303	1331	6539	64.78	20.35	14.86
817	1515	1051	234	2566	59.04	9.12	31.84
901	2134	1685	784	3819	55.88	20.53	23.59
927	3534	2014	1087	5548	63.70	19.59	16.71
829	3932	1994	1165	5926	66.35	19.66	13.99
846	4619	2202	1356	6821	67.72	19.88	12.40
725	6551	2295	1570	8846	74.06	17.75	8.20
673	6887	2760	2087	9647	71.39	21.63	6.98
1017	1374	1136	119	2510	54.74	4.74	40.52
783	3108	1637	854	4745	65.50	18.00	16.50
796	5968	2429	1633	8397	71.07	19.45	9.48
353	8094	2844	2491	10938	74.00	22.77	3.23
901	1844	983	82	2827	65.23	2.90	31.87
734	6248	2694	1960	8942	69.87	21.92	8.21
745	6693	2740	1995	9433	70.95	21.15	7.90
759	5778	2625	1866	8403	68.76	22.21	9.03
1051	2602	1462	411	4064	64.03	10.11	25.86
808	4923	2036	1228	6959	70.74	17.65	11.61
748	7662	3125	2377	10787	71.03	22.04	6.93
1101	1721	1236	135	2957	58.20	4.57	37.23
1144	4506	2382	1238	6888	65.42	17.97	16.61
1080	7023	2893	1813	9916	70.82	18.28	10.89
1377	1946	1590	213	3536	55.03	6.02	38.94
1162	2802	1934	772	4736	59.16	16.30	24.54
977	3474	1694	717	5168	67.22	13.87	18.90

881	3434	2111	1230	5545	61.93	22.18	15.89
1007	3896	2451	1444	6347	61.38	22.75	15.87
830	6955	3223	2393	10178	68.33	23.51	8.15
926	3537	2284	1358	5821	60.76	23.33	15.91
979	3324	2382	1403	5706	58.25	24.59	17.16
751	5713	3078	2327	8791	64.99	26.47	8.54
837	5726	2442	1605	8168	70.10	19.65	10.25
780	8080	3392	2612	11472	70.43	22.77	6.80
750	11134	3307	2557	14441	77.10	17.71	5.19
1032	11465	3443	2411	14908	76.91	16.17	6.92
711	8279	3467	2756	11746	70.48	23.46	6.05
894	8763	3240	2346	12003	73.01	19.55	7.45
876	10939	3110	2234	14049	77.86	15.90	6.24
955	12195	3320	2365	15515	78.60	15.24	6.16
867	6001	2861	1994	8862	67.72	22.50	9.78
636	8451	3210	2574	11661	72.47	22.07	5.45
995	13275	3665	2670	16940	78.36	15.76	5.87
1009	13028	3692	2683	16720	77.92	16.05	6.03
1109	6540	2645	1536	9185	71.20	16.72	12.07
1027	7044	2644	1617	9688	72.71	16.69	10.60
975	11938	3331	2356	15269	78.18	15.43	6.39
1018	12941	3693	2675	16634	77.80	16.08	6.12
1288	2950	3069	1781	6019	49.01	29.59	21.40
967	4563	2173	1206	6736	67.74	17.90	14.36
1068	7360	2624	1556	9984	73.72	15.58	10.70
950	6331	2469	1519	8800	71.94	17.26	10.80
1130	6365	4430	3300	10795	58.96	30.57	10.47
1045	6671	3513	2468	10184	65.50	24.23	10.26
954	10727	3655	2701	14382	74.59	18.78	6.63
1080	11556	4136	3056	15692	73.64	19.47	6.88
948	5517	3505	2557	9022	61.15	28.34	10.51
838	5382	3473	2635	8855	60.78	29.76	9.46
684	9385	3820	3136	13205	71.07	23.75	5.18
805	8854	3842	3037	12696	69.74	23.92	6.34

Table S6. Determination of percentage of hiNeurons (=small nuclei) and hiAstrocytes (=big nuclei) in human NSC-derived CNS cell cultures. hiNeurons were defined as cells with a nuclear area ≤ 100 μm² (small nuclei). hiAstrocytes were defined as cells with a nuclear area > 100 μm² (big nuclei). Three weeks after co-culture, cultures comprised on average 86% hiNeurons (small nuclei) and 14% hiAstrocytes (big nuclei). Data were collected from three (n=3) independently performed experiments acquiring information from approx. 9.8x10⁶ cells. Every row in the table presents acquisition of one complete well of a 96-well-plate. Acquisition and analysis parameters are described in S2 Table. No = number.

No. of cells investigated	% hiNeurons (% small nuclei)	% hiAstrocytes (% big nuclei)	No. of eGFP+ hiAstrocytes	No. of eGFP+ hiNeurons	% eGFP+ hiAstrocytes	% eGFP+ hiNeurons	Biological condition	Treatment
34624	88.52	11.48	0	0	0.00	0.00	non-infected	no treatment
34715	88.29	11.71	7	26	0.02	0.07	non-infected	no treatment
30051	87.22	12.78	1	2	0.00	0.01	non-infected	no treatment
33160	88.69	11.31	0	1	0.00	0.00	non-infected	no treatment
33788	87.50	12.50	1	3	0.00	0.01	non-infected	no treatment
36321	89.07	10.93	0	4	0.00	0.01	non-infected	no treatment
35920	89.24	10.76	0	0	0.00	0.00	non-infected	no treatment
38618	88.67	11.33	0	1	0.00	0.00	non-infected	no treatment
38168	88.52	11.48	13	88	0.03	0.23	non-infected	no treatment
35845	87.33	12.67	0	0	0.00	0.00	non-infected	no treatment
34637	87.22	12.78	0	1	0.00	0.00	non-infected	no treatment

40161	88.03	11.97	2	2	0.00	0.00	non-infected	no treatment
40791	90.92	9.08	1	4	0.00	0.01	non-infected	no treatment
40358	89.97	10.03	1	1	0.00	0.00	non-infected	no treatment
47343	90.37	9.63	14	152	0.03	0.32	non-infected	no treatment
42400	90.42	9.58	0	0	0.00	0.00	non-infected	no treatment
43074	89.41	10.59	0	0	0.00	0.00	non-infected	no treatment
42674	88.22	11.78	0	0	0.00	0.00	non-infected	no treatment
42966	88.93	11.07	2	4	0.00	0.01	non-infected	no treatment
39369	92.01	7.99	0	3	0.00	0.01	non-infected	no treatment
43215	88.93	11.07	0	1	0.00	0.00	non-infected	no treatment
42746	90.06	9.94	0	1	0.00	0.00	non-infected	no treatment
39847	88.27	11.73	2	15	0.01	0.04	non-infected	no treatment
46944	89.61	10.39	0	20	0.00	0.04	non-infected	no treatment
24643	85.10	14.90	2	6	0.01	0.02	non-infected	no treatment
32243	85.98	14.02	0	2	0.00	0.01	non-infected	no treatment
33831	86.67	13.33	0	1	0.00	0.00	non-infected	no treatment
33126	89.51	10.49	0	1	0.00	0.00	non-infected	no treatment
25205	86.09	13.91	0	0	0.00	0.00	non-infected	no treatment
29880	86.21	13.79	0	1	0.00	0.00	non-infected	no treatment

31862	88.45	11.55	0	0	0.00	0.00	non-infected	no treatment
36153	88.31	11.69	0	1	0.00	0.00	non-infected	no treatment
29382	87.79	12.21	0	1	0.00	0.00	non-infected	no treatment
30618	88.52	11.48	1	0	0.00	0.00	non-infected	no treatment
35800	87.70	12.30	0	0	0.00	0.00	non-infected	no treatment
33769	87.58	12.42	0	2	0.00	0.01	non-infected	no treatment
31155	87.27	12.73	2	4	0.01	0.01	non-infected	no treatment
35616	84.32	15.68	0	3	0.00	0.01	non-infected	no treatment
31765	85.20	14.80	1	3	0.00	0.01	non-infected	no treatment
30255	88.22	11.78	4	8	0.01	0.03	non-infected	no treatment
32707	88.26	11.74	0	0	0.00	0.00	non-infected	no treatment
32437	87.33	12.67	0	6	0.00	0.02	non-infected	no treatment
25666	89.09	10.91	0	7	0.00	0.03	non-infected	no treatment
25535	89.48	10.52	0	3	0.00	0.01	non-infected	no treatment
36037	85.59	14.41	0	1	0.00	0.00	non-infected	no treatment
29096	86.09	13.91	3	21	0.01	0.07	non-infected	no treatment
37284	87.20	12.80	1	3	0.00	0.01	non-infected	no treatment
39387	88.39	11.61	0	11	0.00	0.03	non-infected	no treatment
41522	83.86	16.14	10	70	0.02	0.17	non-infected	no treatment

43946	84.91	15.09	1	2	0.00	0.00	non-infected	no treatment
40664	79.83	20.17	0	3	0.00	0.01	non-infected	no treatment
45151	85.36	14.64	10	71	0.02	0.16	non-infected	no treatment
43157	82.86	17.14	1	0	0.00	0.00	non-infected	no treatment
43953	84.20	15.80	4	16	0.01	0.04	non-infected	no treatment
39965	82.69	17.31	0	18	0.00	0.05	non-infected	no treatment
47398	85.06	14.94	0	0	0.00	0.00	non-infected	no treatment
39324	82.57	17.43	0	3	0.00	0.01	non-infected	no treatment
40175	83.40	16.60	1	3	0.00	0.01	non-infected	no treatment
45130	84.21	15.79	0	0	0.00	0.00	non-infected	no treatment
43289	82.56	17.44	0	5	0.00	0.01	non-infected	no treatment
44601	85.25	14.75	6	46	0.01	0.10	non-infected	no treatment
43695	82.84	17.16	0	2	0.00	0.00	non-infected	no treatment
43074	84.11	15.89	0	0	0.00	0.00	non-infected	no treatment
44174	86.59	13.41	2	6	0.00	0.01	non-infected	no treatment
42210	84.45	15.55	1	2	0.00	0.00	non-infected	no treatment
48385	84.23	15.77	0	2	0.00	0.00	non-infected	no treatment
38624	84.18	15.82	0	0	0.00	0.00	non-infected	no treatment
43019	85.22	14.78	1	1	0.00	0.00	non-infected	no treatment

35015	82.31	17.69	1	2	0.00	0.01	non-infected	no treatment
38160	82.25	17.75	0	2	0.00	0.01	non-infected	no treatment
45043	83.56	16.44	0	3	0.00	0.01	non-infected	no treatment
41926	85.07	14.93	1	4	0.00	0.01	non-infected	no treatment
31759	88.88	11.12	3284	15581	10.34	49.06	Th2P-4M-eGFP	IL-1β treatment
42700	88.63	11.37	4681	27048	10.96	63.34	Th2P-4M-eGFP	IL-1β treatment
44834	91.57	8.43	3658	26103	8.16	58.22	Th2P-4M-eGFP	IL-1β treatment
38477	88.04	11.96	4440	23499	11.54	61.07	Th2P-4M-eGFP	IL-1β treatment
31798	90.44	9.56	2840	15255	8.93	47.97	Th2P-4M-eGFP	IL-1β treatment
44829	91.03	8.97	3796	22605	8.47	50.42	Th2P-4M-eGFP	IL-1β treatment
37638	90.49	9.51	3356	20623	8.92	54.79	Th2P-4M-eGFP	IL-1β treatment
34735	86.96	13.04	4139	19335	11.92	55.66	Th2P-4M-eGFP	IL-1β treatment
25263	88.12	11.88	2712	13644	10.74	54.01	Th2P-4M-eGFP	IL-1β treatment
30529	86.81	13.19	3877	20251	12.70	66.33	Th2P-4M-eGFP	IL-1β treatment
26474	83.75	16.25	3999	16522	15.11	62.41	Th2P-4M-eGFP	IL-1β treatment
23069	86.52	13.48	2739	11113	11.87	48.17	Th2P-4M-eGFP	IL-1β treatment
23474	89.52	10.48	2279	14502	9.71	61.78	Th2P-4M-eGFP	IL-1β treatment
30781	86.73	13.27	3984	21748	12.94	70.65	Th2P-4M-eGFP	IL-1β treatment
27244	85.68	14.32	3807	18726	13.97	68.73	Th2P-4M-eGFP	IL-1β treatment

30653	91.08	8.92	2487	15915	8.11	51.92	Th2P-4M-eGFP	IL-1β treatment
38702	83.16	16.84	6417	28027	16.58	72.42	Th2P-4M-eGFP	IL-1β treatment
41782	84.02	15.98	6604	30487	15.81	72.97	Th2P-4M-eGFP	IL-1β treatment
38505	83.83	16.17	6129	27860	15.92	72.35	Th2P-4M-eGFP	IL-1β treatment
37381	81.91	18.09	6576	27139	17.59	72.60	Th2P-4M-eGFP	IL-1β treatment
47160	86.69	13.31	6015	31531	12.75	66.86	Th2P-4M-eGFP	IL-1β treatment
42116	85.69	14.31	5883	29404	13.97	69.82	Th2P-4M-eGFP	IL-1β treatment
29849	83.46	16.54	4810	20279	16.11	67.94	Th2P-4M-eGFP	IL-1β treatment
36968	81.11	18.89	6877	26594	18.60	71.94	Th2P-4M-eGFP	IL-1β treatment
31700	89.45	10.55	3175	17365	10.02	54.78	Th2P-4M-eGFP	IL-6 treatment
35696	90.15	9.85	3411	19642	9.56	55.03	Th2P-4M-eGFP	IL-6 treatment
39146	87.47	12.53	4726	23950	12.07	61.18	Th2P-4M-eGFP	IL-6 treatment
44145	90.63	9.37	3929	24932	8.90	56.48	Th2P-4M-eGFP	IL-6 treatment
28841	90.96	9.04	2357	12891	8.17	44.70	Th2P-4M-eGFP	IL-6 treatment
30568	89.75	10.25	2940	16461	9.62	53.85	Th2P-4M-eGFP	IL-6 treatment
41593	89.80	10.20	3872	21525	9.31	51.75	Th2P-4M-eGFP	IL-6 treatment
39172	90.04	9.96	3631	20404	9.27	52.09	Th2P-4M-eGFP	IL-6 treatment
22492	88.11	11.89	2532	12298	11.26	54.68	Th2P-4M-eGFP	IL-6 treatment
24963	88.64	11.36	2601	13292	10.42	53.25	Th2P-4M-eGFP	IL-6 treatment

31395	87.13	12.87	3796	20935	12.09	66.68	Th2P-4M-eGFP	IL-6 treatment
32602	85.88	14.12	4409	21431	13.52	65.74	Th2P-4M-eGFP	IL-6 treatment
22252	89.20	10.80	2277	12172	10.23	54.70	Th2P-4M-eGFP	IL-6 treatment
23568	90.42	9.58	2039	12761	8.65	54.15	Th2P-4M-eGFP	IL-6 treatment
29444	84.29	15.71	4450	20150	15.11	68.43	Th2P-4M-eGFP	IL-6 treatment
29014	86.96	13.04	3521	17883	12.14	61.64	Th2P-4M-eGFP	IL-6 treatment
29198	83.69	16.31	4655	19661	15.94	67.34	Th2P-4M-eGFP	IL-6 treatment
34896	81.44	18.56	6217	24325	17.82	69.71	Th2P-4M-eGFP	IL-6 treatment
36277	82.57	17.43	6168	26195	17.00	72.21	Th2P-4M-eGFP	IL-6 treatment
41535	83.97	16.03	6494	29288	15.64	70.51	Th2P-4M-eGFP	IL-6 treatment
37141	81.50	18.50	6720	27427	18.09	73.85	Th2P-4M-eGFP	IL-6 treatment
34345	82.06	17.94	6037	24420	17.58	71.10	Th2P-4M-eGFP	IL-6 treatment
43289	87.71	12.29	5229	27764	12.08	64.14	Th2P-4M-eGFP	LIF treatment
33546	89.42	10.58	3417	18681	10.19	55.69	Th2P-4M-eGFP	LIF treatment
38521	88.93	11.07	4057	22860	10.53	59.34	Th2P-4M-eGFP	LIF treatment
35869	87.30	12.70	4400	22029	12.27	61.42	Th2P-4M-eGFP	LIF treatment
37031	89.58	10.42	3669	21057	9.91	56.86	Th2P-4M-eGFP	LIF treatment
30440	88.43	11.57	3265	14766	10.73	48.51	Th2P-4M-eGFP	LIF treatment
40518	89.23	10.77	4002	23331	9.88	57.58	Th2P-4M-eGFP	LIF treatment

37668	88.44	11.56	4094	22567	10.87	59.91	Th2P-4M-eGFP	LIF treatment
29392	87.07	12.93	3673	20262	12.50	68.94	Th2P-4M-eGFP	LIF treatment
24048	86.22	13.78	3053	13684	12.70	56.90	Th2P-4M-eGFP	LIF treatment
23317	84.38	15.62	3304	15006	14.17	64.36	Th2P-4M-eGFP	LIF treatment
25774	84.95	15.05	3633	16614	14.10	64.46	Th2P-4M-eGFP	LIF treatment
37433	81.92	18.08	5598	22449	14.95	59.97	Th2P-4M-eGFP	LIF treatment
20347	87.60	12.40	2241	10181	11.01	50.04	Th2P-4M-eGFP	LIF treatment
26595	84.32	15.68	3838	16374	14.43	61.57	Th2P-4M-eGFP	LIF treatment
21651	85.89	14.11	2933	13926	13.55	64.32	Th2P-4M-eGFP	LIF treatment
28648	80.90	19.10	5311	19275	18.54	67.28	Th2P-4M-eGFP	LIF treatment
31987	84.58	15.42	4826	21174	15.09	66.20	Th2P-4M-eGFP	LIF treatment
35660	83.15	16.85	5912	25964	16.58	72.81	Th2P-4M-eGFP	LIF treatment
38353	82.84	17.16	6457	28023	16.84	73.07	Th2P-4M-eGFP	LIF treatment
32434	86.19	13.81	4421	22171	13.63	68.36	Th2P-4M-eGFP	LIF treatment
33872	80.48	19.52	6441	23697	19.02	69.96	Th2P-4M-eGFP	LIF treatment
39209	82.19	17.81	6591	27665	16.81	70.56	Th2P-4M-eGFP	LIF treatment
41878	84.82	15.18	6235	29633	14.89	70.76	Th2P-4M-eGFP	LIF treatment
34234	88.40	11.60	3713	17409	10.85	50.85	Th2P-4M-eGFP	no treatment
40415	90.27	9.73	3806	24064	9.42	59.54	Th2P-4M-eGFP	no treatment

38689	89.03	10.97	4044	22415	10.45	57.94	Th2P-4M-eGFP	no treatment
43038	90.13	9.87	4134	25059	9.61	58.23	Th2P-4M-eGFP	no treatment
34540	88.83	11.17	3737	20082	10.82	58.14	Th2P-4M-eGFP	no treatment
32006	90.50	9.50	2882	16587	9.00	51.82	Th2P-4M-eGFP	no treatment
34167	86.75	13.25	4408	21480	12.90	62.87	Th2P-4M-eGFP	no treatment
30754	88.40	11.60	3445	17146	11.20	55.75	Th2P-4M-eGFP	no treatment
36717	88.70	11.30	3972	20970	10.82	57.11	Th2P-4M-eGFP	no treatment
36354	89.75	10.25	3588	20525	9.87	56.46	Th2P-4M-eGFP	no treatment
38715	89.60	10.40	3869	22199	9.99	57.34	Th2P-4M-eGFP	no treatment
45365	88.35	11.65	5004	27697	11.03	61.05	Th2P-4M-eGFP	no treatment
21757	86.67	13.33	2757	12500	12.67	57.45	Th2P-4M-eGFP	no treatment
29327	86.33	13.67	3800	17858	12.96	60.89	Th2P-4M-eGFP	no treatment
34736	88.48	11.52	3769	19837	10.85	57.11	Th2P-4M-eGFP	no treatment
31781	87.76	12.24	3566	16666	11.22	52.44	Th2P-4M-eGFP	no treatment
18993	84.68	15.32	2773	11931	14.60	62.82	Th2P-4M-eGFP	no treatment
24065	85.24	14.76	3408	14181	14.16	58.93	Th2P-4M-eGFP	no treatment
26445	86.20	13.80	3391	16397	12.82	62.00	Th2P-4M-eGFP	no treatment
25728	87.11	12.89	3169	15274	12.32	59.37	Th2P-4M-eGFP	no treatment
24506	83.68	16.32	3801	14885	15.51	60.74	Th2P-4M-eGFP	no treatment

25021	86.74	13.26	3227	16771	12.90	67.03	Th2P-4M-eGFP	no treatment
28408	86.45	13.55	3698	19039	13.02	67.02	Th2P-4M-eGFP	no treatment
28594	83.52	16.48	4363	17404	15.26	60.87	Th2P-4M-eGFP	no treatment
41098	85.08	14.92	6023	30309	14.66	73.75	Th2P-4M-eGFP	no treatment
39470	84.30	15.70	6077	27506	15.40	69.69	Th2P-4M-eGFP	no treatment
45023	85.24	14.76	6454	31317	14.33	69.56	Th2P-4M-eGFP	no treatment
46478	88.21	11.79	5364	32071	11.54	69.00	Th2P-4M-eGFP	no treatment
29877	83.56	16.44	4808	20719	16.09	69.35	Th2P-4M-eGFP	no treatment
37063	87.41	12.59	4615	26200	12.45	70.69	Th2P-4M-eGFP	no treatment
32310	84.59	15.41	4875	21998	15.09	68.08	Th2P-4M-eGFP	no treatment
36651	82.59	17.41	6250	25902	17.05	70.67	Th2P-4M-eGFP	no treatment
42089	86.80	13.20	5433	27864	12.91	66.20	Th2P-4M-eGFP	no treatment
40533	86.59	13.41	5351	28190	13.20	69.55	Th2P-4M-eGFP	no treatment
39217	83.94	16.06	6167	28024	15.73	71.46	Th2P-4M-eGFP	no treatment
41887	85.20	14.80	6064	29555	14.48	70.56	Th2P-4M-eGFP	no treatment
33903	89.43	10.57	3012	12287	8.88	36.24	Tha-eGFP	IL-1β treatment
36299	87.96	12.04	3995	16612	11.01	45.76	Tha-eGFP	IL-1β treatment
41511	88.36	11.64	4452	16559	10.72	39.89	Tha-eGFP	IL-1β treatment
33119	87.40	12.60	3769	14869	11.38	44.90	Tha-eGFP	IL-1β treatment

30932	89.56	10.44	2905	11697	9.39	37.82	Tha-eGFP	IL-1β treatment
42388	88.60	11.40	4483	19920	10.58	46.99	Tha-eGFP	IL-1β treatment
41543	87.40	12.60	4842	18023	11.66	43.38	Tha-eGFP	IL-1β treatment
31644	86.97	13.03	3644	15547	11.52	49.13	Tha-eGFP	IL-1β treatment
29375	87.14	12.86	3522	13745	11.99	46.79	Tha-eGFP	IL-1β treatment
32121	84.62	15.38	4722	18266	14.70	56.87	Tha-eGFP	IL-1β treatment
31405	85.86	14.14	4078	16826	12.99	53.58	Tha-eGFP	IL-1β treatment
22689	82.87	17.13	3445	10422	15.18	45.93	Tha-eGFP	IL-1β treatment
25754	86.95	13.05	2793	9085	10.84	35.28	Tha-eGFP	IL-1β treatment
30771	84.29	15.71	4312	14529	14.01	47.22	Tha-eGFP	IL-1β treatment
28463	84.31	15.69	4135	13274	14.53	46.64	Tha-eGFP	IL-1β treatment
23515	83.01	16.99	3515	9443	14.95	40.16	Tha-eGFP	IL-1β treatment
31731	79.41	20.59	6443	19375	20.31	61.06	Tha-eGFP	IL-1β treatment
35216	81.56	18.44	6313	21620	17.93	61.39	Tha-eGFP	IL-1β treatment
34996	82.38	17.62	5927	20458	16.94	58.46	Tha-eGFP	IL-1β treatment
36379	77.02	22.98	7925	21866	21.78	60.11	Tha-eGFP	IL-1β treatment
33383	83.00	17.00	5467	18938	16.38	56.73	Tha-eGFP	IL-1β treatment
33868	79.89	20.11	6006	17309	17.73	51.11	Tha-eGFP	IL-1β treatment
37130	81.44	18.56	6383	21403	17.19	57.64	Tha-eGFP	IL-1β treatment

32254	78.82	21.18	5718	16341	17.73	50.66	Tha-eGFP	IL-1β treatment
29204	87.81	12.19	3336	12561	11.42	43.01	Tha-eGFP	IL-6 treatment
31951	88.11	11.89	3536	14393	11.07	45.05	Tha-eGFP	IL-6 treatment
35869	84.90	15.10	4764	18493	13.28	51.56	Tha-eGFP	IL-6 treatment
42834	90.38	9.62	3847	19610	8.98	45.78	Tha-eGFP	IL-6 treatment
32603	90.01	9.99	3044	14574	9.34	44.70	Tha-eGFP	IL-6 treatment
29989	88.10	11.90	3362	14016	11.21	46.74	Tha-eGFP	IL-6 treatment
35291	86.13	13.87	4484	18548	12.71	52.56	Tha-eGFP	IL-6 treatment
40512	89.08	10.92	4087	18904	10.09	46.66	Tha-eGFP	IL-6 treatment
29152	86.60	13.40	3647	14853	12.51	50.95	Tha-eGFP	IL-6 treatment
24151	86.77	13.23	2984	11512	12.36	47.67	Tha-eGFP	IL-6 treatment
34570	82.05	17.95	5494	19226	15.89	55.61	Tha-eGFP	IL-6 treatment
33384	85.50	14.50	4606	17770	13.80	53.23	Tha-eGFP	IL-6 treatment
22493	83.57	16.43	3266	8846	14.52	39.33	Tha-eGFP	IL-6 treatment
23360	84.33	15.67	3395	10973	14.53	46.97	Tha-eGFP	IL-6 treatment
34162	85.40	14.60	4539	18218	13.29	53.33	Tha-eGFP	IL-6 treatment
32073	86.50	13.50	3959	13940	12.34	43.46	Tha-eGFP	IL-6 treatment
29460	80.89	19.11	5416	17015	18.38	57.76	Tha-eGFP	IL-6 treatment
30949	81.41	18.59	5406	16722	17.47	54.03	Tha-eGFP	IL-6 treatment

33839	78.72	21.28	6775	19771	20.02	58.43	Tha-eGFP	IL-6 treatment
36815	84.87	15.13	5349	21115	14.53	57.35	Tha-eGFP	IL-6 treatment
33980	82.89	17.11	5374	18210	15.82	53.59	Tha-eGFP	IL-6 treatment
27094	83.13	16.87	4195	13918	15.48	51.37	Tha-eGFP	IL-6 treatment
36110	77.72	22.28	6790	20189	18.80	55.91	Tha-eGFP	IL-6 treatment
39336	81.01	18.99	6501	21504	16.53	54.67	Tha-eGFP	IL-6 treatment
42906	90.36	9.64	3864	19022	9.01	44.33	Tha-eGFP	LIF treatment
32247	88.49	11.51	3453	14087	10.71	43.68	Tha-eGFP	LIF treatment
34731	85.57	14.43	4534	16431	13.05	47.31	Tha-eGFP	LIF treatment
34335	85.61	14.39	4350	17049	12.67	49.65	Tha-eGFP	LIF treatment
42767	89.66	10.34	4188	20434	9.79	47.78	Tha-eGFP	LIF treatment
28511	86.97	13.03	3474	12897	12.18	45.24	Tha-eGFP	LIF treatment
35825	82.86	17.14	5046	16944	14.09	47.30	Tha-eGFP	LIF treatment
34972	85.38	14.62	4501	17224	12.87	49.25	Tha-eGFP	LIF treatment
30255	84.58	15.42	4473	17004	14.78	56.20	Tha-eGFP	LIF treatment
27468	87.11	12.89	3288	12940	11.97	47.11	Tha-eGFP	LIF treatment
28260	83.13	16.87	4474	16555	15.83	58.58	Tha-eGFP	LIF treatment
30257	84.95	15.05	4309	16524	14.24	54.61	Tha-eGFP	LIF treatment
30344	88.48	11.52	3266	13771	10.76	45.38	Tha-eGFP	LIF treatment

23660	85.25	14.75	3173	10087	13.41	42.63	Tha-eGFP	LIF treatment
29269	85.21	14.79	3674	13654	12.55	46.65	Tha-eGFP	LIF treatment
30682	82.63	17.37	4629	15762	15.09	51.37	Tha-eGFP	LIF treatment
32398	83.56	16.44	5204	19110	16.06	58.99	Tha-eGFP	LIF treatment
33159	83.94	16.06	5051	17046	15.23	51.41	Tha-eGFP	LIF treatment
34573	79.70	20.30	6202	19056	17.94	55.12	Tha-eGFP	LIF treatment
36503	81.28	18.72	6499	21710	17.80	59.47	Tha-eGFP	LIF treatment
35245	82.18	17.82	6023	20747	17.09	58.87	Tha-eGFP	LIF treatment
30724	82.75	17.25	5033	15969	16.38	51.98	Tha-eGFP	LIF treatment
36705	82.26	17.74	5916	21767	16.12	59.30	Tha-eGFP	LIF treatment
39121	81.03	18.97	6748	21958	17.25	56.13	Tha-eGFP	LIF treatment
30073	87.88	12.12	3155	11557	10.49	38.43	Tha-eGFP	no treatment
37850	89.09	10.91	3891	16569	10.28	43.78	Tha-eGFP	no treatment
41244	88.98	11.02	4161	18850	10.09	45.70	Tha-eGFP	no treatment
36567	90.01	9.99	3153	13709	8.62	37.49	Tha-eGFP	no treatment
29012	87.94	12.06	3080	11078	10.62	38.18	Tha-eGFP	no treatment
31334	85.27	14.73	4100	14926	13.08	47.64	Tha-eGFP	no treatment
29509	86.20	13.80	3837	14036	13.00	47.57	Tha-eGFP	no treatment
28002	84.47	15.53	4028	12521	14.38	44.71	Tha-eGFP	no treatment
32911	87.88	12.12	3688	14575	11.21	44.29	Tha-eGFP	no treatment
34034	87.25	12.75	3938	15480	11.57	45.48	Tha-eGFP	no treatment
31329	86.05	13.95	3973	13776	12.68	43.97	Tha-eGFP	no treatment
32642	86.63	13.37	3983	15815	12.20	48.45	Tha-eGFP	no treatment
22316	83.42	16.58	3518	12355	15.76	55.36	Tha-eGFP	no treatment
30318	86.28	13.72	3868	15250	12.76	50.30	Tha-eGFP	no treatment
27786	86.68	13.32	3393	13226	12.21	47.60	Tha-eGFP	no treatment
33471	87.89	12.11	3809	17542	11.38	52.41	Tha-eGFP	no treatment
28824	86.07	13.93	3820	14875	13.25	51.61	Tha-eGFP	no treatment

26236	86.73	13.27	3219	12504	12.27	47.66	Tha-eGFP	no treatment
27351	87.03	12.97	3312	12980	12.11	47.46	Tha-eGFP	no treatment
23144	82.54	17.46	3625	9028	15.66	39.01	Tha-eGFP	no treatment
32984	86.56	13.44	4155	17206	12.60	52.16	Tha-eGFP	no treatment
33616	85.45	14.55	4546	17935	13.52	53.35	Tha-eGFP	no treatment
29048	83.26	16.74	4617	15628	15.89	53.80	Tha-eGFP	no treatment
31363	85.95	14.05	4093	16977	13.05	54.13	Tha-eGFP	no treatment
31400	81.03	18.97	5806	18698	18.49	59.55	Tha-eGFP	no treatment
31023	82.20	17.80	5344	18599	17.23	59.95	Tha-eGFP	no treatment
33463	82.80	17.20	5493	19122	16.42	57.14	Tha-eGFP	no treatment
38768	84.35	15.65	5865	20450	15.13	52.75	Tha-eGFP	no treatment
32832	84.62	15.38	4688	17042	14.28	51.91	Tha-eGFP	no treatment
30430	84.81	15.19	4357	16248	14.32	53.39	Tha-eGFP	no treatment
31173	83.99	16.01	4699	16853	15.07	54.06	Tha-eGFP	no treatment
37071	84.58	15.42	5499	19882	14.83	53.63	Tha-eGFP	no treatment
33219	81.82	18.18	5823	18286	17.53	55.05	Tha-eGFP	no treatment
30851	82.33	17.67	5329	18457	17.27	59.83	Tha-eGFP	no treatment
29498	81.10	18.90	5199	15132	17.62	51.30	Tha-eGFP	no treatment
29550	77.84	22.16	6312	17306	21.36	58.57	Tha-eGFP	no treatment
